# A Reconfigurable Memristive Spiking Neuron Enabling Advanced Neuromorphic Computing

**DOI:** 10.1002/advs.77375

**Published:** 2026-08-24

**Authors:** Pek Jun Tiw, Yuqi Li, Zhongyuan Li, Qihang Ding, Yuzhe Wang, Jiarong Wang, Yuchao Yang

**Affiliations:** ^1^ New Cornerstone Science Laboratory Beijing Advanced Innovation Center for Integrated Circuits School of Integrated Circuits Peking University Beijing China; ^2^ New Cornerstone Science Laboratory Guangdong Provincial Key Laboratory of In‐Memory Computing Chips School of Electronic and Computer Engineering Shenzhen Graduate School Peking University Shenzhen China; ^3^ Center for Brain Inspired Chips Institute For Artificial Intelligence Peking University Beijing China; ^4^ Center for Brain Inspired Intelligence Chinese Institute For Brain Research (CIBR) Beijing China

**Keywords:** adaptation, bursting, fast‐spiking, neural coding, reconfigurable, single‐spiking, tunability

## Abstract

Biological neurons exhibit internal complexity that enables a variety of spiking behaviors, complex encoding strategies, and the possibility of advanced networks that underpin cognitive brain functions. Neuromorphic computing, especially using memristors with biologically plausible dynamics, is imperative to realizing next‐generation artificial intelligence. However, current attempts still rely on simple neurons with limited support for novel network algorithms. This work proposes a multi‐mode reconfigurable memristive spiking neuron with a simple transistor‐capacitor feedback circuit that enables fast‐spiking, adaptive spiking, phasic bursting, or single‐spiking. Every spike train parameter in each mode can be freely tuned. With this, a multiplexed encoding strategy in a hazard avoidance application is demonstrated, wherein 3 driving actions and 6 maneuvering control variables are encoded using distinct spiking modes and spike train parameters. Furthermore, long‐short‐term memory spiking neural networks (LSNN) that incorporate either heterogeneous slow time constants or dynamic mode switching are proposed, demonstrating up to 8.0% and 13.7% improvements in accuracy in highly temporal tasks compared with homogeneous LSNNs, while also exhibiting superior generalization capabilities. The ability to support these advanced encoding and network strategies distinguishes the proposed reconfigurable neuron from existing implementations, highlighting its potential to empower more advanced neuromorphic computing.

## Graphical abstract

Pek Jun Tiw and co‐workers develop a reconfigurable memristive neuron that can exhibit four spiking behaviors with freely tunable spike train parameters. The neuron supports multiplexed spike encoding of multiple pieces of information. It also supports advanced neural network strategies, such as neural heterogeneity and dynamic mode switching, achieving temporal task accuracy improvements of up to 8.0% and 13.7%, respectively.

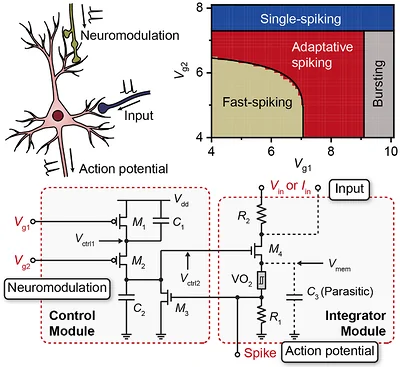

## 1 Introduction

The brain operates on a rich underlying substrate of structures, including different types of neurons and neuronal projections [1, 2]. Emerging from this substrate are various strategies, such as multiplexed neural coding [3, 4], neural heterogeneity [5, 6, 7], and dynamic regime switching [8], which support the fine‐scale encoding of disparate information, functional network diversity, and dynamic adaptation of spiking mode for specific cognitive behaviors. The enhanced internal complexity of neurons and the external complexity of large‐scale topologically‐rich networks together enable the brain to perform complex temporal processing at high efficiency [9]. These computing principles are fundamentally different from those employed in conventional computing paradigms and most brain‐inspired algorithms, such as deep‐learning artificial neural networks [10, 11, 12, 13, 14, 15]. On the other hand, neuromorphic computing seeks to emulate powerful yet efficient brain computations at the hardware level by utilizing parallel event‐driven computing primitives [16, 17]. This paradigm is crucial when faced with ever‐increasing computing demands for artificial intelligence.

To this end, various mathematical models of neurons have been proposed, ranging from the simplified leaky integrate‐and‐fire (LIF) model to the bio‐plausible Hodgkin‐Huxley (HH) model [18]. However, emulating these neurons on conventional hardware platforms is inefficient [19, 20, 21]. Extending the notion of neuromorphic to the device level entails using emerging devices with neurobiology‐like physical properties, such as memristors [22, 23]. The memristive LIF neuron has been successfully utilized in many spiking neural network (SNN) applications [24, 25, 26, 27, 28]. Nevertheless, they lack the computational power of biological neurons, let alone forming the basis of more computationally‐capable networks. Strategies to augment it with complex dynamics for stronger computing capabilities are constantly being pursued. These include rediscovering neuronal behaviors such as adaptation for enhanced temporal processing [29, 30, 31, 32], and bursting [33, 34, 35, 36], which pertains to attention [37]. Another strategy involves incorporating temporal coding schemes that are inherently fast and efficient [38, 39, 40], which underpin many rapid biological responses [41, 42]. Yet, only a few works have reported modified memristive LIF implementations that support multiple spiking behaviors, but with coding parameters within complex spiking modes often being coupled to each other [33, 36], thus limiting the degree of freedom that the neuron can rely on to express complex information. Memristive HH neurons have also been proposed [43, 44], which endow simple neural circuits with intelligent sensory behaviors [34]. However, switching between spiking modes in these neurons requires changing circuit elements, which is not feasible for large‐scale integration.

At the network level, several works have shown that networks of heterogeneous memristive neurons can exhibit innate processing capabilities [34, 45]. Nonetheless, the level of heterogeneity possible is still limited because current demonstrations still rely on integrating multiple specifically engineered, non‐reconfigurable neurons, and implementing a large collection of such neurons while maintaining a certain degree of algorithmic reconfigurability wastes chip real estate. Besides, networks with spatial or temporal organization can be constructed using simple memristive neurons with multiterminal inputs [46, 47, 48]. However, biologically plausible SNNs with higher complexity and spatiotemporal processing capabilities, such as those incorporating multifunctional neuromodulatory inputs, have not yet been reported.

Here, we seek to enhance the computational complexity of the LIF neuron while ensuring tunability. Using a VO_2_ threshold switching volatile memristor, we propose a multi‐mode reconfigurable neuron that exhibits fast‐spiking, single‐spiking, phasic bursting, and adaptation, whereby the spiking regime is controlled by a transistor‐capacitor feedback module with dynamics subjected to two control biases and the input stimulus itself. We explore the parameter space defined by these inputs to verify the reconfigurability of the neuron and to evaluate the tunability of the spike train coding parameters within each regime. Using the reconfigurability and tunability of the neuron, we demonstrate a multiplexed encoding strategy in a hazard‐avoidance scenario, achieving the encoding of 3 discrete driving actions and 6 control variables in a single output channel. We further demonstrate a heterogeneous long‐short‐term memory SNN (LSNN) that incorporates heterogeneous adaptation and a dynamic LSNN that supports the dynamic evolution of the spiking regime of neurons within, showing up to 8.0% and 13.7% improvements in accuracy, respectively, on temporal tasks alongside better generalization capabilities. This highlights the possibility of constructing biologically plausible SNN algorithms with superior processing power using our reconfigurable neuron and its potential role in more advanced neuromorphic computing systems.

## 2 Result and Discussion

### 2.1 Bio‐Plausible Neuromodulation With Reconfigurable Neurons

Figure 1a depicts a simplified illustration of neuromodulation, which is ubiquitous in the brain [1]. For example, in pyramidal neurons, apical tuft inputs may control the firing behavior of the neuron in response to proximal inputs [49]. Such mechanisms couple with distinct morphologies of different neurons to enable a variety of spiking dynamics, providing the brain with a rich repertoire of neuronal behaviors for performing computations. Inspired by such structural heterogeneity, we propose constructing a reconfigurable neuron using an integrator‐control modular design. The integrator module, based on a threshold switching memristor, specifically a VO_2_ Mott memristor in our case, receives inputs that act directly on the membrane potential to generate spikes. On the other hand, the control module comprising a transistor‐capacitor network that receives two modulatory inputs alters the membrane dynamics of the integrator and configures the neuron's working mode. Depending on the biasing scheme, our neuron can operate in four distinct regimes: fast‐spiking, single‐spiking, phasic bursting, and adaptive spiking (Figure 1b). Each regime can be further characterized by one or more freely tunable spike train parameters. These properties enable the emulation of more complex computational substrates with enhanced internal and external complexity, such as multiplexed spike coding, neural heterogeneity, and dynamic neural mode (Figure 1c). In this work, multiplexed spike coding is used in a hazard avoidance task under the general context of robotic control and involves the representation of a general action using a spiking mode alongside the exact control variables or states of that action using spike train parameters. Neural heterogeneity involves configuring a network through external biasing such that the temporal dynamics of the neurons within is different from each other. Dynamic neural mode involves allowing the operating regime of neurons within the network to dynamically change depending on the input and the current state of the network. The details of the implementation of these computational substrates will be provided in the subsequent sections.

**FIGURE 1:**
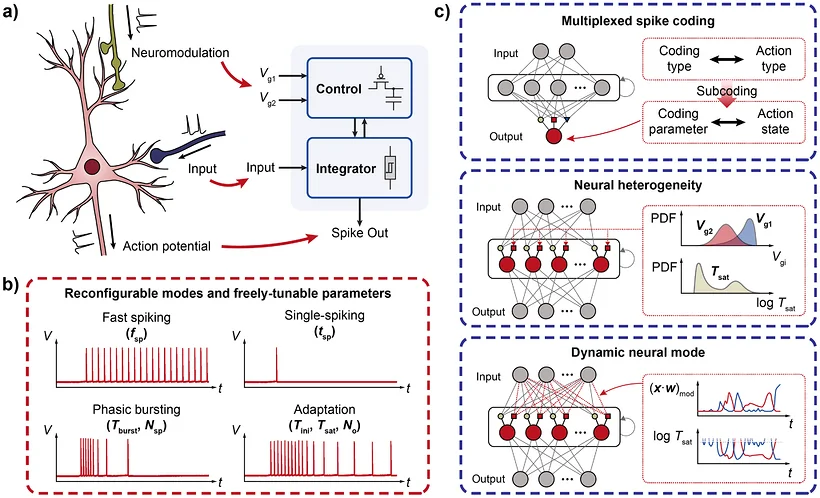
A biologically plausible multi‐mode reconfigurable neuron for next‐generation neuromorphic computing. (a) A correspondence between an exemplary biological neuron and a module‐level schematic of our proposed reconfigurable neuron. Biological neurons receive functionally specialized presynaptic inputs, such as local excitatory inputs and neuromodulatory projections, and output action potentials with rich dynamics. Our neuron consists of an integrator and a control module, respectively receiving one excitatory input and two modulatory inputs. (b) The four spiking modes or regimes achievable by our neuron through port biasing, each with a different number of freely tunable spike train parameters. (c) Examples of biologically plausible SNN strategies explored in this work such as multiplexed spike coding, neural heterogeneity, and dynamic neural mode switching.

### 2.2 Reconfigurable Neuron Design and Biasing Scheme

We demonstrate our neuron using a VO_2_ Mott memristor for its scalability [50], high‐speed operation [51], CMOS compatibility [52], and superior uniformity [38] to better highlight the properties of the neuron circuit as a whole. We fabricated planar VO_2_ devices, as depicted in the device structure model and the scanning electron micrograph (SEM) in Figure 2a, following the procedures detailed in the Experimental section. Figure 2b plots the *I*–*V* characteristics of the VO_2_ memristor. The interplay between Joule heating and nonlinear material properties [53] results in abrupt threshold switching behaviors at *V*_th, VO2_ and *V*_hold, VO2_ under quasi‐static voltage sweeps, and a negative differential resistance under quasi‐static current sweeps. These properties enable threshold comparison, spontaneous reset, and self‐oscillation dynamics, all of which are key to constructing spiking neurons. Note that other volatile threshold switching memristors, such as the NbO*_x_* Mott memristor [36] and diffusive memristors [54], can also work as they exhibit similar electrical characteristics. Nonetheless, the exact material, mechanism, or device does not substantially affect subsequent discussions on circuit design and operation.

**FIGURE 2:**
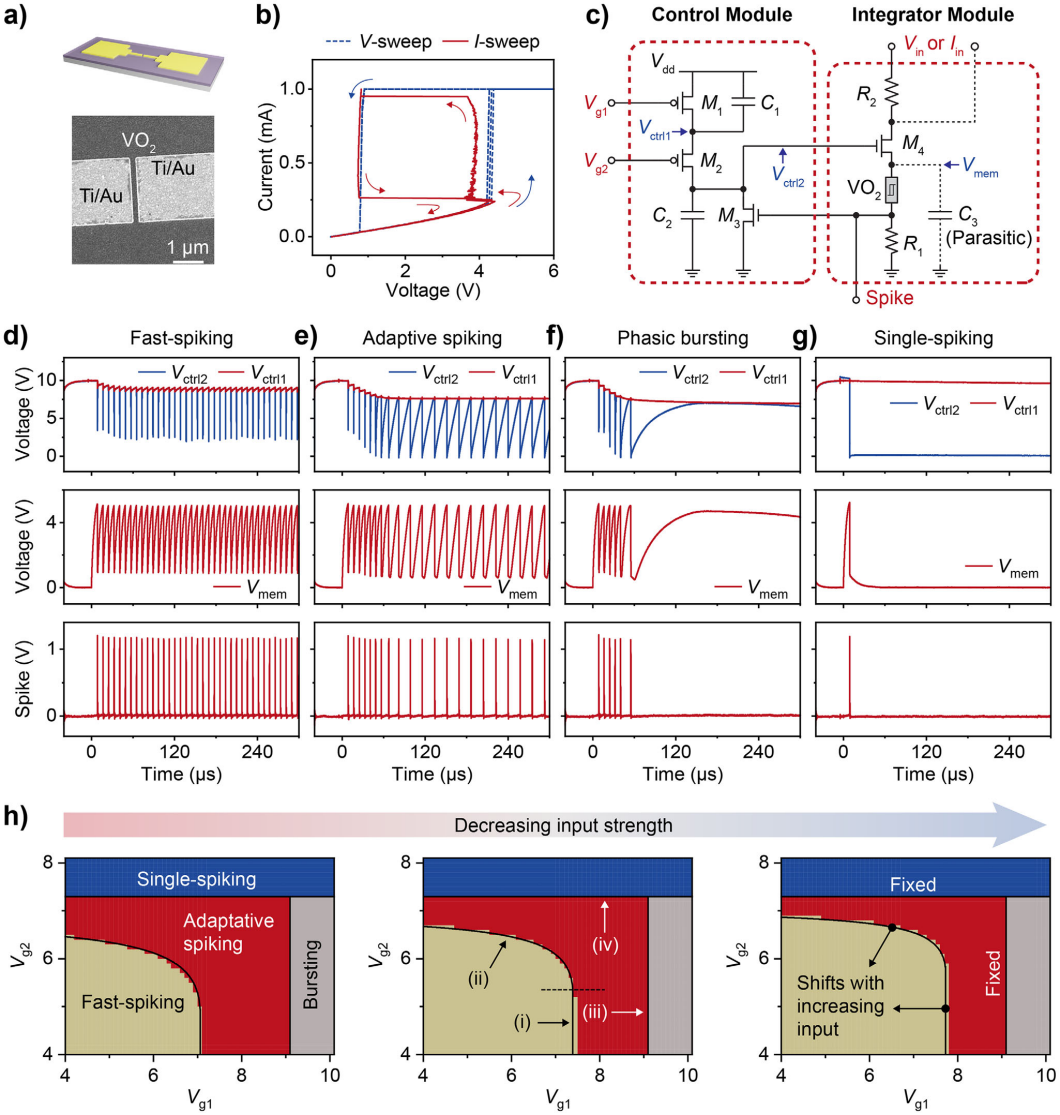
Reconfigurable spiking neuron design and its multiple spiking regimes. (a) A model and an SEM image of the VO_2_ memristor used to demonstrate our neuron design. (b) The quasi‐static *I*–*V* curves of the VO_2_ memristor. (c) Circuit schematic of the proposed reconfigurable neuron in which a control module regulates an integrator module via a feedback pathway. (d–g) Evolution of *V*_ctrl1_, *V*_ctrl2_, *V*_mem_, and the spike output of the neuron in the (d) fast‐spiking, (e) adaptive spiking, (f) phasic bursting, and (g) single‐spiking regimes. (h) Phase diagrams as functions of *V*_g1_ and *V*_g2_, showing regions of spiking regimes. *V*_in_ decreases from left to right. Black solid lines indicated by (i–iv) denote the boundaries between regimes defined by the mathematical models in Equations (1), (2), (3), (4) respectively. *V*_in_ shifts the boundary between fast‐spiking and adaptive spiking. The boundaries between adaptive spiking, phasic bursting, and single‐spiking stay fixed regardless of *V*_in_, *V*_g1_, and *V*_g2_.

Various spiking modes can be regarded as an extension of the commonly implemented LIF‐based fast‐spiking. To enable reconfigurability between spiking modes, we designed a simple yet easily tunable transistor‐capacitor feedback control module that receives spikes from the integrator module and, in turn, gates the integrator input via an n‐type pass transistor *M*_4_, thereby achieving dynamical control over its excitability (Figure 2c). Initially, *V*_ctrl1_ and *V*_ctrl2_ are high, turning on *M*_4_ and allowing the leaky integration of input signals on the membrane capacitance *C*_3_. When *V*_mem_ reaches *V*_th, VO2_, the memristor switches to the low‐resistance state, and the integrator module fires a spike. The spike discharges *C*_2_ via *M*_3_ and decreases *V*_ctrl2_ by some amount Δ*V*_sp_. Meanwhile, *M*_1_, *M*_2_ (under *V*_g1_ and *V*_g2_ biases), and *C*_1_ control the recharging speed of *C*_2_ and the equilibrium behavior of *V*_ctrl1_ and *V*_ctrl2_. In general, *V*_ctrl1_ and *V*_ctrl2_ show a decreasing trend with each spike. Under these competing factors, the neuron eventually settles into one of the four spiking modes as exemplified by the experimentally measured responses to a constant integrator input in Figure 2d–g. The respective behaviors of *V*_ctrl1_ and *V*_ctrl2_ are also shown. Additionally, Figures S1 and S2 show the simulated response of the neuron under a periodic input pulse train and a random input pulse train, respectively. Using experimentally extracted device and circuit parameters, we performed simulations by sweeping over the parameter space defined by *V*_g1_, *V*_g2_, and *V*_in_ to reveal the transitions between active operating regimes as depicted in the phase diagrams in Figure 2h. Also plotted in the phase diagrams are mathematically derived boundaries, which are presented below. The derivations of the boundaries are given in Note S1 while the algorithmic approach used to obtain Figure 2h is given in Note S2.

To enable fast‐spiking (Figure 2d), *V*_g1_ and *V*_g2_ should be biased sufficiently low such that both *M*_1_ and *M*_2_ are fully turned on. This ensures that *V*_ctrl1_ only fluctuates at a higher voltage without dipping too low and maintains the current through *M*_2_, |*I*_ds,_ *_M_*_2_|, so that *V*_ctrl2_ follows *V*_ctrl1_ closely. Hence, *M*_4_ is also fully on, providing an unimpeded path for input stimuli to accumulate on *C*_3_. The circuit behaves effectively as a typical LIF neuron.

To bias the neuron in the adaptive spiking regime (Figure 2e), the inequality *T*_sat_ > *T*_ini_ must hold, where *T*_ini_ and *T*_sat_ denote the initial spiking period and the post‐adaptation saturation spiking period, respectively. The boundary between fast‐spiking and adaptation can be split into two parts, denoted by (i) and (ii), as shown in Figure 2h. They can be described by Equations 1 and 2, respectively.

(1) $$T_{\mathrm{sat}}=\frac{C_{2}\Delta V_{\mathrm{sp}}}{\left|I_{\mathrm{ds},M1,\mathrm{sat}}\left(V_{g1}\right)\right|}=T_{\mathrm{ini}}$$

(2) $$T_{\mathrm{sat}}=\frac{C_{2}\Delta V_{\mathrm{sp}}}{\left|I_{\mathrm{ds},M2,\mathrm{sat}}\left(V_{g1},\;V_{g2}\right)\right|}=T_{\mathrm{ini}}$$

|*I*_ds,_ *_M_*_1, sat_(*V*_g1_)| is the saturation current through *M*_1_, which is a function of *V*_g1_ only. |*I*_ds,_ *_M_*_2, sat_(*V*_g1_, *V*_g2_)| is the saturation current through *M*_2_, which is a function of both *V*_g1_ and *V*_g2_. Adaptation is achieved by ultimately limiting |*I*_ds,_ *_M_*_2_|, that is, by increasing *V*_g1_, *V*_g2_, or both. On one hand, if *V*_g2_ is high enough, *V*_gs,_ *_M_*_2_ becomes sufficiently small to limit |*I*_ds,_ *_M_*_2_| after the circuit fires a few spikes. On the other hand, if *V*_g1_ is high enough, |*I*_ds,_ *_M_*_1_| is limited and *V*_ctrl1_ decreases considerably with each spike, eventually reducing *V*_gs,_ *_M_*_2_ sufficiently such that |*I*_ds,_ *_M_*_2_| also becomes limited. Consequently, the low |*I*_ds,_ *_M_*_2_| prolongs the charging of *C*_2_. Maintaining the required current through *M*_4_, *I*_ds,_ *_M_*_4_, requires *V*_mem_ to rise slowly with *V*_ctrl2_, resulting in an increased spiking period manifested as spike frequency adaptation. Note that *T*_ini_ depends on the input strength received by the integrator. Hence, the boundary *T*_sat_ = *T*_ini_ shifts with the input strength, specifically toward lower *V*_g1_ and *V*_g2_ when the input strength increases.

For phasic bursting (Figure 2f), *M*_1_ must be turned off, and *V*_g2_ must be low enough to turn *M*_2_ on for at least a few spikes. The boundary between phasic bursting and adaptive spiking, denoted by (iii) in Figure 2h, corresponds to the transistor threshold of *M*_1_ (Equation 3).

(3) $$V_{g1}=V_{\mathrm{dd}}\;-\left|V_{\mathrm{th},M1}\right|$$

Under this biasing scheme, *C*_1_ cannot be charged, and *V*_ctrl1_ decreases monotonically with each spike. After firing several spikes, *V*_gs,_ *_M_*_2_ becomes insufficient for *M*_2_ to charge *C*_2_, causing *V*_ctrl2_ to dwell at a level too low to maintain the *I*_ds,_ *_M_*_4_ needed to charge *V*_mem_ to *V*_th, VO2_. Hence, the neuron ceases being excited by any input stimuli after firing a few spikes and enters a resting state.

The neuron transitions into the single‐spiking regime if *V*_g2_ exceeds a specific threshold, regardless of *V*_g1_ (Figure 2g). This threshold corresponds to boundary (iv) in Figure 2h, which is given by Equation 4.

(4) $$V_{g2}=V_{\mathrm{dd}}\;-\left|V_{\mathrm{th},M2}\right|-\frac{C_{2}}{C_{1}}\left(V_{\mathrm{ctrl}2,\mathrm{th}}+\Delta V_{\mathrm{sp}}-V_{\mathrm{dd}}\right)$$

(5) $$V_{\mathrm{ctrl}2,\mathrm{th}}=V_{\mathrm{th},\mathrm{VO}2}+V_{\mathrm{th},M4}+\sqrt{\frac{2V_{\mathrm{th},\mathrm{VO}2}}{R_{\mathrm{VO}2}K_{M4}}}$$

Equation 5 defines *V*_ctrl2, th_, which is the value of *V*_ctrl2_ just enough for *V*_mem_ to reach *V*_th, VO2_. After firing one spike, *C*_2_ is sufficiently discharged, and *M*_4_ turns off. A *V*_g2_ larger than Equation 4 ensures that *M*_2_ turns off before *V*_ctrl2_ can increase enough to turn *M*_4_ on. Therefore, the neuron fires only one spike and then remains in the resting state. One requirement is that Δ*V*_sp_ must be large enough. Otherwise, the neuron needs to fire more than one spike before idling.

As neuromorphic computing progresses toward large scale integration, a few important aspects that require serious consideration when designing neurons include the area overhead, scalability, and power consumption. In‐depth analyses in these areas are given in Note S3 (Tables S1 and S2). Another key aspect when a neuron design involves emerging devices is the effect of variations in these devices on workings of the neuron. In our case, the effect of *V*_th, VO2_ variations on the operating regime boundaries are discussed in Note S4 (Figures S3 and S4).

### 2.3 Freely Tunable Neural Coding Parameters

To highlight the tunability of the proposed reconfigurable neuron for neural coding, we performed relevant electrical characterizations and simulations. Circuit parameters used in the simulations are fitted to the experimentally measured device and circuit behaviors (Table S1). We also provide mathematical models describing the coding behavior below, along with their detailed derivations in Note S1.

The constant spiking frequency, *f*_sp_, in the fast‐spiking regime facilitates conventional rate coding. Under a constant input stimulus, *f*_sp_ is the sole tunable parameter. While circuit parameters such as input resistance, membrane capacitance, and environmental factors such as temperature can modulate *f*_sp_ [25], here we consider only tuning *f*_sp_ via the amplitude of *V*_in_. As *V*_in_ increases, *C*_3_ charges faster, and *V*_mem_ requires less time to reach *V*_th, VO2_. Although the subsequent discharging process becomes slower, its duration is generally negligible compared to the charging process. Hence, as shown in Figure 3a and Figure S5, *f*_sp_ increases with *V*_in_. Information is encoded in *f*_sp_ based on the mathematical model described by Equations (6), (7), (8).

(6) $$t_{r}=R_{\mathrm{eff},\mathrm{off}}\;C_{3}\cdot \ln\left(\frac{\beta_{\mathrm{off}}V_{\mathrm{in}}-V_{\mathrm{hold},\mathrm{VO}2}}{\beta_{\mathrm{off}}V_{\mathrm{in}}-V_{\mathrm{th},\mathrm{VO}2}}\right)$$

(7) $$t_{f}=R_{\mathrm{eff},\mathrm{on}}\;C_{3}\cdot \ln\left(\frac{V_{\mathrm{th},\mathrm{VO}2}-\beta_{\mathrm{on}}V_{\mathrm{in}}}{V_{\mathrm{hold},\mathrm{VO}2}-\beta_{\mathrm{on}}V_{\mathrm{in}}}\right)$$

(8) $$f_{\mathrm{sp}}=\frac{1}{t_{r}+t_{f}}$$

*t*_r_ and *t*_f_ denote the charging time and discharging time, respectively. *R*_eff_ is the effective parallel resistance formed by (*R*_VO2_ + *R*_1_) and the input load resistance, *β* is a voltage divider factor for node *V*_mem_, and the subscript “on” or “off” represents the corresponding term calculated when the VO_2_ memristor is in its low‐resistance state or high‐resistance state.

**FIGURE 3:**
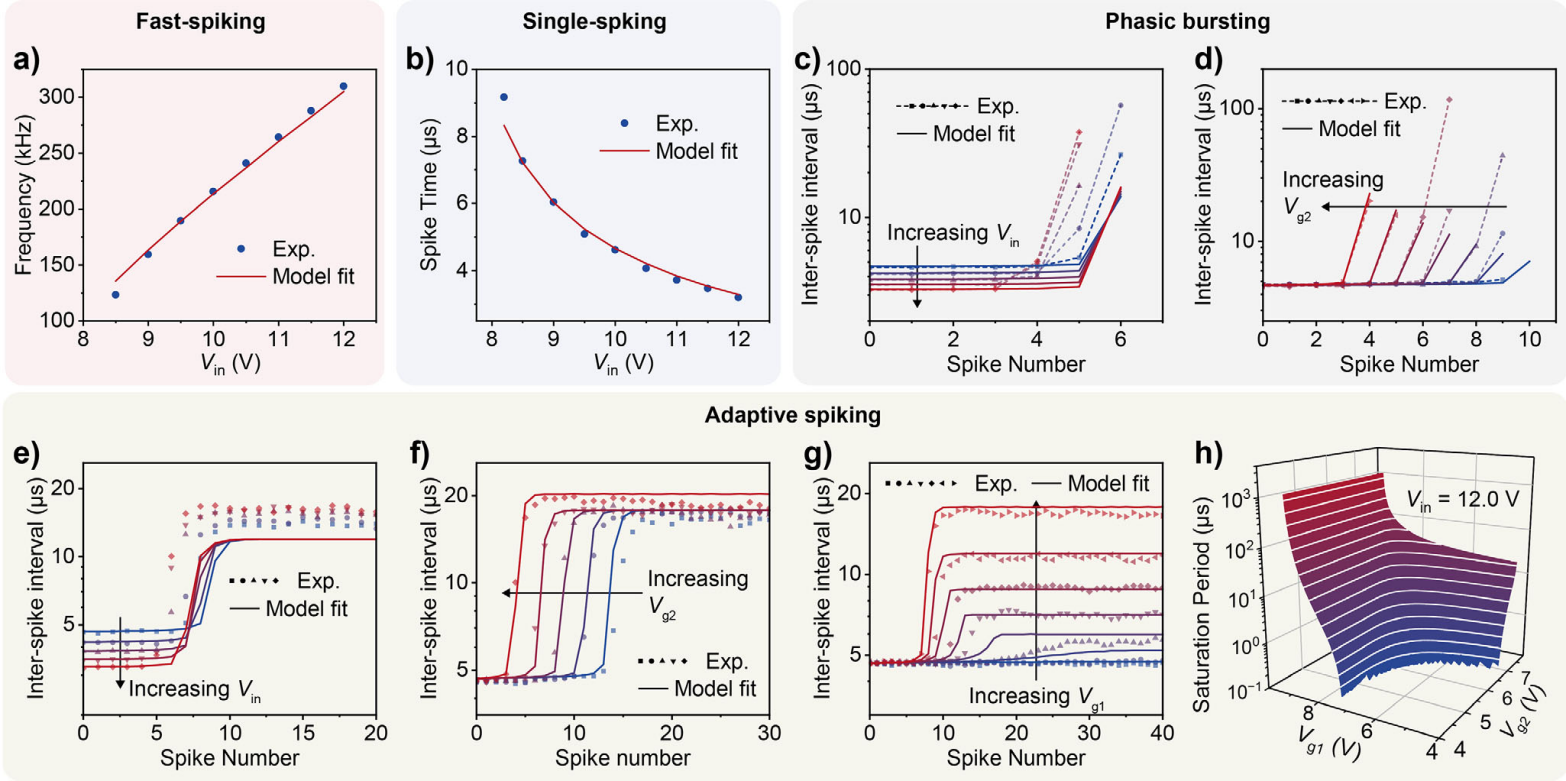
The freely tunable spike train parameters of our neuron. (a) Modulation of spiking frequency via *V*_in_ in the fast‐spiking regime, enabling rate coding with one parameter. (b) Modulation of time‐to‐spike in the single‐spiking regime, enabling single‐spike coding with one parameter. (c,d) Modulation of (c) bursting period via *V*_in_ and (d) total spike count via *V*_g2_, enabling burst coding with two independent parameters. (e–g) Modulation of (e) initial spiking period via *V*_in_, (f) onset of adaptation via *V*_g2_, and (g) saturation spiking period via *V*_g1_. (h) Dependence of saturation spiking period on *V*_g1_ and *V*_g2_. When biased at low *V*_g2_, *V*_g1_ provides a wide tuning range, almost unaffected by *V*_g2_. This enables adaptive rate coding with three freely tunable parameters.

Single‐spike coding embeds information in the timing of the sole spike, *t*_sp_, from the onset of the input, and is the only coding parameter. As shown in Figure 3b and Figure S6, *t*_sp_ can be easily tuned via *V*_in_ because *V*_in_ affects the charging of *C*_3_, similar to rate coding but with superior speed and energy efficiency [38]. *V*_g1_ and *V*_g2_ are only used to bias the neuron in this regime, playing no role in modulating the coding parameter. The relationship is given by Equation 9.

(9) $$t_{\mathrm{sp}}=R_{\mathrm{eff},\mathrm{off}}\;C_{3}\cdot \ln\left(\frac{\beta_{\mathrm{off}}V_{\mathrm{in}}}{\beta_{\mathrm{off}}V_{\mathrm{in}}-V_{\mathrm{th},\mathrm{VO}2}}\right)$$

Burst coding can be characterized by two parameters, the intra‐burst spiking period, *T*_burst_, and the number of burst spikes, *N*_sp_. As shown in Figure 3c and Figure S7, *T*_burst_ is determined by *V*_in_, just as in rate coding under the fast‐spiking regime. On the other hand, *N*_sp_ is modulated only by *V*_g2,_ as shown in Figure 3d and Figure S8, and the relationship is given by Equation 10.

(10) $$N_{\mathrm{sp}}=\lceil \frac{1}{\Delta V_{\mathrm{sp}}}\left[\frac{C_{1}}{C_{2}}\left(V_{\mathrm{dd}}-V_{g2}-\left|V_{\mathrm{th},M2}\right|\right)+V_{\mathrm{dd}}-V_{\mathrm{ctrl}2,\mathrm{th}}\right]\rceil$$

As mentioned in the previous section, the neuron stops firing when *V*_ctrl1_ decreases sufficiently such that *V*_ctrl2_ cannot reach *V*_ctrl2, th_. This condition is met when |*V*_gs, *M*2_| = *V*_ctrl1_ – *V*_g2_< |*V*_th, *M*2_|. Combined with the conservation of charge, Equation 10 can be derived. Another observation is that *T*_burst_ and *N*_sp_ are mutually independent, providing the flexibility needed to encode two separate pieces of information in a single spike train.

In adaptive rate coding, the aforementioned *T*_ini_ and *T*_sat_, together with the onset of adaptation, *N*_o_, can each carry information. Therefore, adaptive rate coding has three parameters. First, similar to *T*_burst_ and *f*_sp_, *T*_ini_ is modulated by *V*_in_. This relationship can be observed in Figure 3e and Figure S9. Second, *N*_o_ can be roughly defined as the number of initial spikes fired before the inter‐spike interval (ISI) starts to increase. This parameter is similar to *N*_sp_ in burst coding and can be defined by Equation 11.

(11) $$N_{o}=\lceil \frac{C_{1}\left(V_{\mathrm{dd}}-V_{g2}-\left|V_{\mathrm{th},M2}\right|\right)+C_{2}\left(V_{\mathrm{dd}}-V_{\mathrm{ctrl}2,\mathrm{th}}\right)}{C_{2}\Delta V_{\mathrm{sp}}-T_{\mathrm{ini}}\left|I_{\mathrm{ds},M1}\right|}\rceil$$

As shown in Figure 3f and Figure S10, *N*_o_ is mainly modulated by *V*_g2_. Before adaptation, a larger *V*_g2_ reduces the charging strength of *C*_2_ between spikes. Thus, *V*_ctrl2_ decreases at a faster rate, resulting in an earlier onset of adaptation. Lastly, *T*_sat_ is modulated by *V*_g1_, as shown in Figure 3g and Figure S11. This relationship is given by the first equality in Equation 1 above, regardless of the operating regime of *M*_2_, when *M*_1_ is in saturation. A larger *V*_g1_ lowers the post‐adaptation equilibrium value of *V*_ctrl1_, which in turn decreases |*I*_ds,_ *_M_*_2_| and increases the charging time of *C*_2_. Thus, *T*_sat_ increases. Further analysis reveals that *V*_g2_ also affects *T*_sat_ (Figure 3h) if *M*_1_ is in the linear regime and *M*_2_ is saturated (first equality in Equation 2). However, Figure 3h also shows that *V*_g1_ can modulate *T*_sat_ in a wider range within a larger biasing interval, making it a better tuning knob. Besides, when *V*_g2_ is not too large, its effect on *T*_sat_ is small. Thus, biasing the neuron in this region ensures maximum freedom when tuning the parameters. A total of three distinct pieces of information can be multiplexed into one spike train.

As can be seen, the neuron relies on stable and accurate evolution of *V*_ctrl1_ and *V*_ctrl2_, which can be affected by various hardware nonidealities such as *V*_g1,2_ noise, *V*_dd_ noise, CMOS process variations, VO_2_ memristor variations, short‐channel effects, parasitic effects, and so forth. In‐depth analyses of the effects of these nonidealities on spike coding are given in Note S5 and Figures S12–S15.

### 2.4 Multiplexed Spike Coding

Biological neurons, such as those found in the thalamus [4] or the cerebellar cortex [3], often encode multiple pieces of information via multiplexing. This is achieved in two ways, that is, by encoding distinct information via different firing modes, such as bursting and tonic firing, and via different properties of a particular spiking mode, such as burst spike count, burst duration, and so forth. Such an encoding paradigm requires multi‐mode reconfigurability and freely tunable spike train parameters, both of which are baked into our neuron design. Here, we demonstrate a multiplexed spike coding strategy using our reconfigurable neuron.

We exemplify this by considering a simplified hazard‐avoidance scenario, in which one car (car A, ego vehicle) is approaching a second car (car B, hazard) ahead that is decelerating. Car A needs to decide on what driving actions to take and the corresponding control variables based on the relative radial distance (*r*) and the azimuth angle (*θ*) to avoid a potential collision (Figure 4a). First, discrete driving actions can each be assigned to distinct coding schemes for a physically compact representation. As described in Figure 4b, we can assign single‐spiking, phasic bursting, and adaptive spiking to throttling back, braking, and steering while braking, respectively. The system should throttle back when it is approaching car B slowly and does not see an immediate threat, brake when it is nearing fast but is laterally separated by some distance, and steer while braking when collision is imminent. Second, multiple control variables per action can each be encoded by one of many coding parameters, enabling multiplexed information representation. We opt to represent control variables such as the throttle position by *t*_sp_, the target post‐braking speed with and without steering by *T*_burst_ and *T*_ini_, respectively, the braking strength with and without steering by *N*_sp_ and *N*_o_, respectively, and the steering angle by *T*_sat_ for a more fine‐grained control. Figure 4c further visualizes the mapping from scenario to actions and maneuvering control variables to *V*_g1_ and *V*_g2_ using the phase diagram of operating regimes. This results in a total of 3 coding regimes and 6 coding parameters multiplexed into a single spiking channel.

**FIGURE 4:**
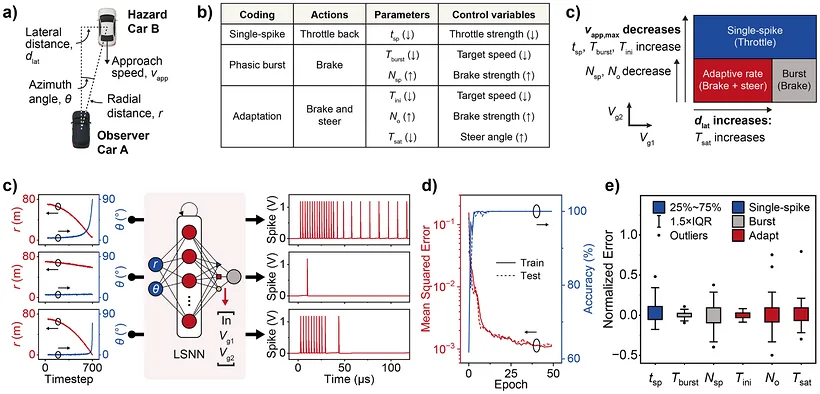
Multiplexed output spike encoding for hazard avoidance. (a) A collision avoidance setting in which the ego vehicle decides which driving actions to take and how to perform the maneuver based on *r* and *θ*. (b) Mapping of driving actions and control variables to spike coding types and spike train parameters. (c) Relationship between scenario (characterized by *d*_lat_ and *v*_app, max_), driving actions, spike train parameters, and *V*_g1,2_ visualized using the phase diagram of spiking regimes. Ground truth driving actions and states are determined by *d*_lat_ and *v*_app, max_. (d) Schematic of the network with one multiplexed output channel and examples of input‐output pairs. (e) Evolution of the loss and accuracy during training. (f) Normalized error of spike train encoding parameters attained after training. Low errors were achieved, indicating accurate multiplexed encoding of actions and control variables.

We generated our own custom dataset by computing *r* and *θ* as a function of time, given an initial *r*_0_, a random lateral distance, *d*_lat_, and a random deceleration of the vehicle ahead, *a* (Experimental section). To relate to real‐world applications such as self‐driving, this effectively emulates the simplest input data that can be derived from on‐board sensors, such as light detection and ranging (LiDAR) scanners. Arbitrary values of *d*_lat_ and the maximum rate of change of *r*, *v*_app, max_, were chosen as thresholds for when to throttle back, brake only, or brake and steer. These further translate to the boundaries between the three operating regimes of the output neuron. We trained a long short‐term SNN (LSNN) [55] with only one reconfigurable output neuron on this task (Figure 4d). All hidden neurons are similarly biased within the adaptive regime. The output neuron can be viewed as having three independent inputs, each receiving a weighted sum of activations from the hidden layer. The spiking regime and spike train parameters of the output neuron are to be learned. Task performance was evaluated using the mean squared error (MSE) between the inferred and target combinations of the integrator input strength, *V*_g1_ and *V*_g2_, that correspond to the required spike train output. The results are shown in Figure 4e,f, wherein the output neuron generated driving actions with 100% accuracy and minimal errors across the multiplexed coding parameters for the control variables. Such a network with a multiplexed coding strategy is also robust against various hardware nonidealities (Note S6 and Figure S16).

As summarized in Table S2, our neuron surpasses existing implementations in terms of the tunability of coding parameters and the number of multiplexed coding regimes and sub‐coding states demonstrated. Owing to the more accurate emulation of how the biological nervous system encodes information, the proposed neuron and its natural fit for multiplex encoding help bridge the gap between neuromorphic computing and neuroscience.

### 2.5 Networks With Heterogeneous and Dynamic Slow Time Constants

Cortical neurons exhibit heterogeneity in neural time constants, which aids in processing complex tasks [5, 56]. Such heterogeneity can be effectively captured by our reconfigurable neuron thanks to its tunable control module. Here, we propose two types of LSNNs that leverage neural heterogeneity in static and dynamic manners, respectively, to process temporal information with high performance (Figure 5a). These networks can be implemented using our proposed neuron.

**FIGURE 5:**
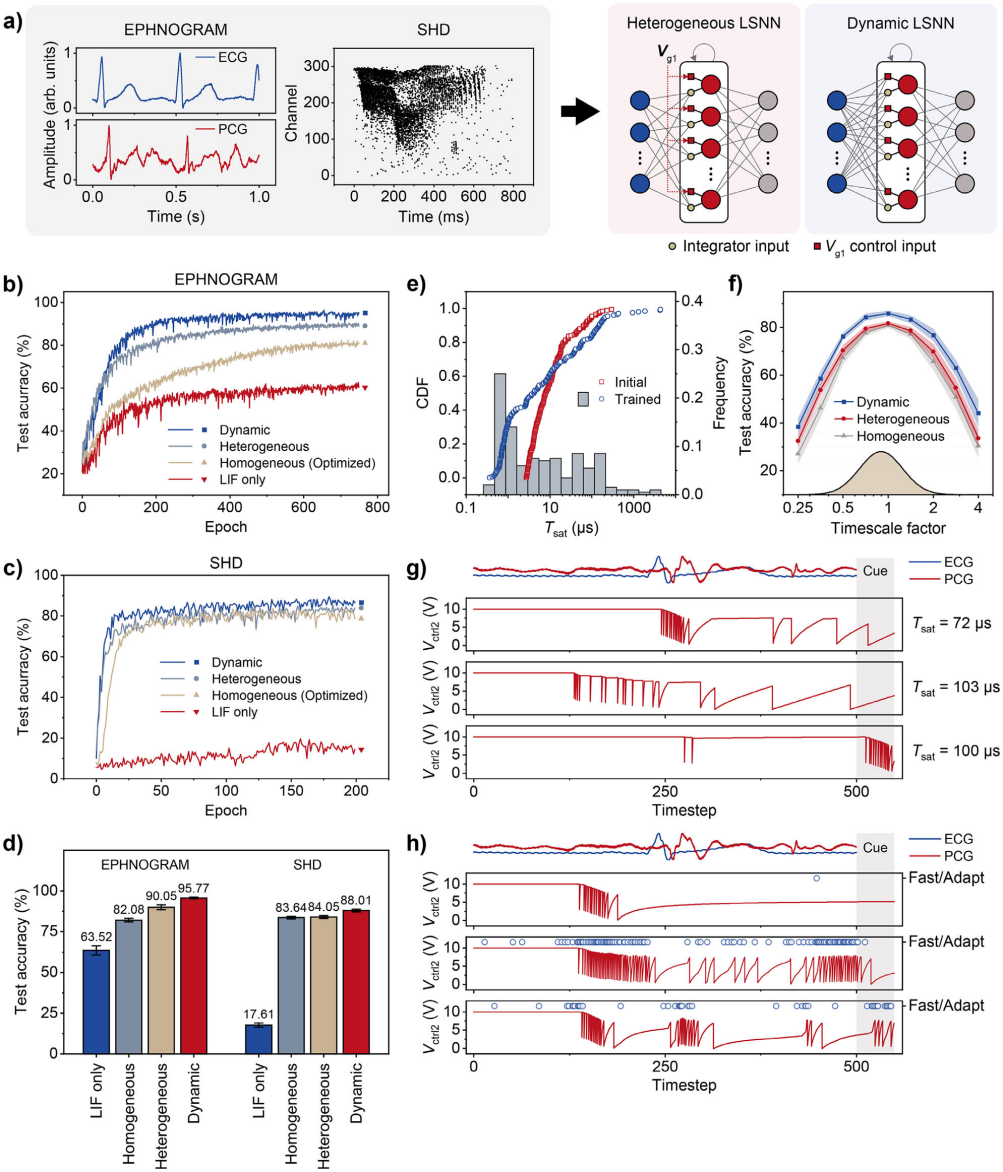
Biologically plausible SNNs with improved performance on temporal datasets. (a) Typical input samples from the EPHNOGRAM and SHD datasets, and a schematic of the heterogeneous LSNN and dynamic LSNN. In the heterogeneous LSNN, hidden‐layer neurons are configured with a fixed vector **V**_g1_ to spike adaptively with different slow time constants. In the dynamic LSNN, hidden‐layer neurons receive weighted inputs at their *V*_g1_ ports. *V*_g2_ biasing is fixed in both cases and is therefore omitted to prevent clutter. (b,c) The evolution of test accuracies of LIF‐only, homogeneous, heterogeneous, and dynamic LSNNs on the (b) EPHNOGRAM and (c) SHD datasets. (d) Comparison of the maximum test accuracies between the four LSNNs on both datasets, averaged over *n* = 6 randomly initialized trials. Error bars represent ±1 standard deviation from the mean. (e) Redistribution of *T*_sat_ of a heterogeneous LSNN with trainable *V*_g1_ biases. (f) Network test accuracies on the SHD dataset under fixed timescale factors, after training with factors sampled from the lognormal distribution shown. (g) *V*_ctrl2_ evolution of three hidden neurons in the heterogeneous LSNN illustrating negative imprinting: active (inactive) neurons before the cue phase fire with difficulty (ease) during the cue phase. (h) *V*_ctrl2_ evolution of three hidden neurons in the dynamic LSNN, showing both negative imprinting and selective response to specific input features.

In the static case (termed “Heterogeneous LSNN”), neurons in the hidden recurrent layer are biased in the adaptive spiking regime and pre‐configured with different *T*_sat_ via the tuning knob *V*_g1_. Note that characterizing neurons using *T*_sat_ is equivalent to using the adaptive slow time constant to some extent. *V*_g1_ can be sampled from a given distribution and stay fixed throughout the inference process. *V*_g2_ can be either fixed to the same value for all neurons or to different values sampled from a distribution to achieve greater heterogeneity. Note that the *T*_sat_ heterogeneity considered here differs from the membrane time constant (*τ*_m_) heterogeneity typically considered in other works [5, 7]. In memristive spiking neurons, *τ*_m_ depends on the membrane capacitance and the high‐resistance state of the memristor, both of which are difficult to adjust post‐fabrication. While it is possible to introduce additional variable resistive elements in the spike‐generating pathway to tune *τ*_m_, this might alter the circuit biasing and sacrifice reconfigurability and tunability.

In the dynamic case (termed “Dynamic LSNN”), the spiking mode of the neuron evolves dynamically. If the neuron is in the adaptive spiking regime, *T*_sat_ can also evolve dynamically. To achieve this, we configured *V*_g1_ as a second input port for each neuron, allowing it vary based on the linearly weighted sum of input activations (Equation 12).

(12) $$V_{g1}\;\left(t\right)=f\left(x\left(t\right)W_{f}+y\left(t\right)W_{r}\right)$$

**x**(*t*) and **y**(*t*) are activation vectors from the preceding and current layers, **W**_f_ and **W**_r_ are feed‐forward and recurrent weight matrices that feed into the control module. *f*(∙) is included for the sake of completeness as it represents a placeholder function for transforming the weighted sum to a usable control voltage and depends on the exact hardware implementation. Thus, the spiking regime and *T*_sat_, if applicable, depend on the current input stimuli and network states. While *V*_g2_ can also be configured to evolve dynamically, we set it to be fixed in our experiments.

As controls, we also included a LIF‐only LSNN and a homogeneous adaptive LIF‐only (ALIF) LSNN (termed “Homogeneous LSNN”). In the LIF‐only LSNN, all hidden neurons are biased in the fast‐spiking regime. In the homogeneous LSNN, all hidden neurons are instead biased in the adaptive regime with a single optimized *T*_sat_ value. The optimized *T*_sat_ was chosen to achieve the highest classification accuracy.

We evaluate the networks on the electro‐phono‐cardiogram (EPHNOGRAM) dataset [57, 58] and the Spiking Heidelberg Digits (SHD) dataset [59], each having different underlying temporal structures. The EPHNOGRAM dataset records complementary electrocardiogram (ECG) and phonocardiogram (PCG) signals from the heart during rest and physical activities at varying intensities. The networks are tasked with categorizing 1 s clips of recordings into five physical scenarios: sitting in an armchair, lying on a bed, walking at a constant speed, cycling, and performing treadmill stress. The SHD dataset consists of spike events that represent the frequency spectrum of spoken digits, generated by passing auditory signals through a cochlear model. Information is tightly coupled both spatially and temporally. The networks are tasked with categorizing the 10 digits spoken in English and German.

The accuracies of the four networks on the EPHNOGRAM and SHD test datasets over training epochs are shown in Figure 5b,c, respectively. The maximum test accuracy achieved for each network type is averaged over *n* = 6 randomly initialized trials and summarized in Figure 5d. Homogeneous LSNNs of ALIF neurons perform better than LIF‐only LSNNs, which is consistent with the results reported in our previous work [29]. Networks of ALIF neurons compute via the negative imprinting principle [60], where neurons actively spiking (inactive) before the output phase indicated by a “CUE” signal are harder (easier) to excite during the output stage. LIF‐only networks lack this property and thus perform poorly on highly temporal datasets.

Heterogeneous LSNNs perform generally better than homogeneous LSNNs. However, the magnitude of improvement is greater on the EPHNOGRAM task than on the SHD task due to the different underlying temporal structure. At least two different scales of temporality exist within the EPHNOGRAM recordings, in that information is present between the shorter relative times of feature occurrences within and between the ECG and PCG signals, which capture the interrelationship between the electrical and mechanical properties of the heart, and the longer period between successive beats, which is described by the heart rate. On the contrary, spoken digits are non‐periodic, and information is distributed within the short time it takes to say a word. Thus, the EPHNOGRAM task could benefit more from using a wider range of time constants to process multi‐timescale information. *T*_sat_ can also be learned by setting *V*_g1_ to be trainable. However, we found that training *T*_sat_ does not result in a significant improvement in accuracy, at least for the two datasets considered (Figure S17). Figure 5e shows the initial and trained distributions of *T*_sat_ of the neurons in the SHD task. After redistribution, about 15% of the neurons have a *T*_sat_ greater than 100 µs, which is comparable to the input duration of 240 µs. This fraction agrees well with that of experimentally observed adaptive neurons in the neocortex (Figure S18) [61].

Nevertheless, the dynamic LSNN, which is possible only by adopting our neuron, achieves the best performance on both datasets. Similar to homogeneous or heterogeneous LSNNs, dynamic LSNNs also exhibit negative imprinting. This is evident in the evolution of *V*_ctrl2_ in heterogeneous and dynamic LSNNs, as shown in Figure 5g,h, respectively. Additional evolutions of *V*_ctrl2_ in response to different input samples are given in Figure S19. When the network learned weights that result in a high *V*_g1_ throughout the input duration, the neuron is always in the phasic bursting regime or the adaptive regime with a large *T*_sat_, and it behaves similarly to the ALIF neurons in a conventional LSNN. However, there also exist weights that temporarily drive some neurons into the fast‐spiking regime or the adaptive regime with a small *T*_sat_, enabling the neuron to restore its excitability and respond to multiple features in the inputs. This allows neurons to react to a wide range of input feature combinations, thereby expanding the feature maps of the hidden layer for superior spatiotemporal processing capabilities. Treating the control port as a separate neuron input introduces additional trainable parameters, bringing the total to almost twice that of a non‐dynamic LSNN. Thus, it is reasonable to attribute the increased performance to the larger parameter space. However, we performed additional experiments and observed that a heterogeneous LSNN with a wider hidden layer to match the number of trainable parameters cannot achieve the same level of performance as a dynamic LSNN on both datasets (Figure S17). This indicates that the dynamical switching between regimes does, in fact, introduce extra computing capabilities beyond negative imprinting. We would also like to point out that these networks, especially the dynamic LSNN, exhibit robustness against hardware nonidealities. An analysis of the effect of nonidealities on network performance is provided in Note S6 and Figures S20 and S21.

In certain cases, signals can span different timescales but still carry the same information. For example, different speaking paces do not significantly change the meaning of words or phrases in voice command recognition. Thus, the network in such systems must be able to generalize across timescales. We investigated how homogeneous, heterogeneous, and dynamic LSNNs perform under varying timescales on the SHD dataset (Figure 5f). Similar to the augmentation strategy in ref [5], we scaled the input spike times by a factor randomly sampled from a lognormal distribution (*µ* = 0, *σ* = 0.33) for each batch during training. The networks (*n* = 6 for each network type) were then tested on specific timescale factors ranging between 0.25 × to 4 ×. Other network parameters remained the same. Heterogeneous LSNNs achieve the same peak performance as optimized homogeneous LSNNs but can generalize better to other timescales. This trend is consistent with results reported when considering the absence or presence of *τ*_m_‐heterogeneity [5], indicating the effectiveness of *T*_sat_‐homogeneity. On the other hand, dynamic LSNNs perform the best across all timescales, once again substantiating their superior temporal computing capabilities.

As summarized in Table S2, the ability of our neuron to fully support more complex SNNs, such as heterogeneous and dynamic LSNNs, with improved performance distinguishes it from existing implementations. It is worth noting that such a neuron with multi‐mode reconfigurability and ease of tunability is not limited to realizing only the networks demonstrated here. In fact, it opens up possibilities for other network structures with substantial extrinsic complexity. We also envision the proposed neuron to form the basis for future neuromorphic systems, in which a collection of organized multifunctional network modules intercommunicate and collaborate to perform advanced computing tasks, akin to how multiple areas of the brain interconnect and contribute to the emergence of complex cognitive behaviors [62].

## 3 Conclusion

Reproducing the brain's computational abilities on neuromorphic hardware demands innovation at multiple levels, including neuron circuit design, encoding strategies, and network algorithms. We have introduced a multi‐mode reconfigurable spiking neuron based on volatile threshold switching memristors that can switch between fast‐spiking, phasic bursting, adaptive spiking, and single‐spiking modes depending on the biasing scheme. The ability to freely tune spike train parameters in each mode was demonstrated. Using this feature, we demonstrated a multiplexed encoding strategy in a hazard‐avoidance scenario by representing discrete information with 3 spiking modes in a single channel and fine‐scale information with 6 spike train parameters. Additionally, we demonstrated that the proposed neuron supports biologically plausible LSNNs that include adaptation timescale neural heterogeneity or dynamic mode switching. These unconventional SNNs exhibited performance improvements of up to 8.0% and 13.7%, respectively, on temporal tasks and better generalization compared to commonly used networks of either homogeneous adaptive LIF neurons or vanilla LIF neurons. This reconfigurable, freely tunable neuron opens the door to implementing novel SNN algorithms, potentially bridging the gap between neuromorphic computing and neuroscience and paving the way for advanced artificial intelligence.

## 4 Experimental Section

### 4.1 VO_2_ Devices

Epitaxial VO_2_ thin films were grown on polished *c*‐Al_2_O_3_ substrates via molecular beam epitaxy. During deposition, the substrate temperature was held at 550°C. Metallic vanadium was electron‐beam evaporated from a vanadium powder source at a rate of 0.1 Å s^−1^ under an oxygen flux of 2.5 s.c.c.m. while maintaining a chamber pressure of 1–3 mPa. The evaporation rate was monitored using a crystal oscillator. Activated oxygen was provided by a standard radio‐frequency plasma source, and the flow rate was controlled by a 0.1 s.c.c.m. precision mass flow controller. The deposition process was monitored in situ using reflective high‐energy electron diffraction. After deposition, a final post‐annealing step was carried out under a high vacuum at 300°C for 2 h. The VO_2_ devices were fabricated by patterning the electrodes using electron‐beam lithography, followed by electron‐beam evaporation of a 10 nm Ti adhesion layer and 100 nm Au contacts and lift‐off.

### 4.2 Electrical Measurements

All electrical measurements were performed using a semiconductor parameter analyzer (Agilent B1500A) and a digital storage oscilloscope (Keysight DSOS404A) whenever necessary. Connections from external circuitries to the VO_2_ memristor were made using a probe station (FormFactor Cascade Summit200). The experimental setup is shown in Figure S22.

### 4.3 Custom Hazard‐Avoidance Dataset

In this dataset, the input signals are the time evolutions of *r* and *θ*, and the target output is a spike train defined by a specific combination of *I*_in_, *V*_g1_, and *V*_g2_. The time evolutions of *r* and *θ* were computed over 700 timesteps by using Equation 13 and the discretized version of Equation 14.

(13) $$\theta =\arcsin\left(\frac{d_{\mathrm{lat}}}{r}\right)$$

(14) $$\frac{d}{dt}r=\left(v_{0}-at\right)\;\sqrt{1-{\left(\frac{d_{\mathrm{lat}}}{r}\right)}^{2}}$$

*v*_0_ is a fixed initial relative velocity. *d*_lat_ and *a* were randomly generated to obtain 1000 samples. Arbitrary thresholds *d*_lat, th_ and *v*_app, th_ were set as the boundaries between driving actions, where *v*_app_ = Δ*r*/Δ*t*. The target output is single‐spiking if *v*_app, max_ < *v*_app, th_, phasic bursting if *v*_app, max_ ≥ *v*_app, th_ and *d*_lat_ > *d*_lat, th_, and adaptation if *v*_app, max_ ≥ *v*_app, th_ and *d*_lat_ ≤ *d*_lat, th_, where *v*_app, max_ is the maximum *v*_app_ over time for each sample. The fine‐scale sub‐coding strategies of control variables are defined by the linear mappings in Equation (15), (16), (17).

(15) $$I_{\mathrm{in}}=I_{\mathrm{in},\min}+\left(I_{\mathrm{in},\max}-I_{\mathrm{in},\max}\right)\cdot \left(\frac{v_{\mathrm{app},\max}-v_{\min}}{v_{\max}-v_{\min}}\right)$$

(16) $$V_{g1}=V_{g1,\min}+\left(V_{g1,\max}-V_{g1,\min}\right)\cdot \frac{d_{\mathrm{lat}}}{d_{l\mathrm{at},\max}}$$

(17) $$V_{g2}=V_{g2,\min}+\left(V_{g2,\max}-V_{g2,\min}\right)\cdot \left(\frac{v_{\mathrm{app},\max}-v_{\mathrm{app},\mathrm{th}}}{v_{\max}-v_{\mathrm{app},\mathrm{th}}}\right)$$

where *I*_in, min_, *I*_in, max_, *V*_g1, min_, *V*_g1, max_, *V*_g2, min_ and *V*_g2, max_ are the minimum and maximum input current and control voltages, *v*_min_ and *v*_max_ are the minimum and maximum relative velocity over time across all samples, *d*_lat, max_ is the maximum lateral distance.

### 4.4 EPHNOGRAM Dataset

The EPHNOGRAM dataset contains records with varying signal quality [57, 58]. Poor quality records, as labeled in the spreadsheet summary accompanying the dataset, were removed. The remaining records were categorized into 5 classes: sitting in an armchair, lying on a bed, walking at a constant speed, cycling, and performing treadmill stress. The ECG and PCG channels of each record were resampled to 2 kHz and split into 1 s segments. This resulted in a variable number of heartbeats per segment, with each beat occurring at different temporal positions relative to the start of the segment. Each segment was normalized along the time dimension. After preprocessing all records, 2000 instances of 1 s segments from each class were randomly sampled to form a final dataset of 10 000 samples. The dataset was randomly split into a training and a testing subset with sizes of 7936 and 2064, respectively. Each sample was finally encoded as spike trains using the asynchronous delta encoding strategy proposed in our previous work [29]. In essence, each analog input channel was converted into two spike trains (UP and DOWN). A spike event was generated in the UP or DOWN channel whenever the input signal amplitude increased or decreased by a fixed value *δ* = 0.05.

### 4.5 SHD Dataset

The SHD dataset comprises utterances of digits with varying durations [59]. To limit the input time duration, spikes occurring after 0.8 s were dropped for each input sample. This involves 24.88% and 22.26% of the training and testing samples, respectively, but accounts for only 0.19% and 0.11% of all spikes in the respective subsets. The remaining spike events were binned into 1 ms bins. The spatial dimension of each sample was downsampled for augmentation purposes by randomly splitting the original 700 channels into 2 sets of 350 each, thereby doubling the dataset size. Further augmentation was applied by introducing spike time jitter and spike channel jitter.

### 4.6 SNN Simulations

The LSNNs in this work are based on SNNs with a recurrent hidden layer comprising a mix of adaptive and non‐adaptive spiking neurons [55]. In all networks, a cue signal was appended to the end of the input sample to signal the output phase. The output activation vector during the final timestep was used to determine the output. In the hazard‐avoidance task, the loss function was defined as the mean squared error between the generated and target *I*_in_, *V*_g1_, and *V*_g2_. In the EPHNOGRAM and SHD classification tasks, the categorical cross‐entropy loss was used instead.

The networks were implemented using the PyTorch‐based SpikingJelly deep SNN learning framework [63]. Backpropagation through time (BPTT) with surrogate gradients was used to train the networks [64]. The inverse tangent function (Equation 18) was used to approximate the gradients.

(18) $$\begin{array}{c} f\;\left(x\right)=\frac{1}{\pi^{2}}\;\arctan\left(\pi x\right) \end{array}$$

To speed up network simulations, the input spike trains were reduced along the time dimension. Spikes were further binned into larger timesteps, and a weighted sum of the binned spikes based on their relative positions within the larger timestep was performed (Equation 19). This resulted in graded spikes ensuring consistent neuron dynamics.

(19) $$\begin{array}{c} \;S_{i}=\sum_{j=1}^{N}s_{j}\exp\left[-\frac{\Delta t/N}{\tau_{m}}\cdot \left(N-j\right)\right] \end{array}$$

Δ*t* is the larger timestep duration, *τ*_m_ is the membrane time constant, *N* is the reduction factor (Δ*t* is equally divided into *N* smaller timesteps), *s_j_* is the spike at the *j*‐th position within Δ*t*, and *S_i_* is the graded spike at the *i*‐th timestep in the final graded spike train. *N* = 4 was used for both the EPHNOGRAM and SHD classification tasks.

All relevant network parameters are listed in Table S3.

## Author Contributions

**Yuqi Li**: investigation. **Yuzhe Wang**: investigation. **Jiarong Wang**: investigation. **Yuchao Yang**: writing – review and editing, funding acquisition, supervision. **Qihang Ding**: investigation. **Pek Jun Tiw**: conceptualization, writing – original draft, investigation, validation, formal analysis. **Zhongyuan Li**: investigation.

## Conflicts of Interest

The authors declare no conflict of interest.

## Supporting information




**Supporting File**: advs77375‐sup‐0001‐SuppMat.docx.

## Data Availability

The data that support the findings of this study are available from the corresponding author upon reasonable request.
