# Supplementary material for: A Reconfigurable Memristive Spiking Neuron Enabling Advanced Neuromorphic Computing
Source: Adv Sci (Weinh). 2026 Aug 24;13(63):e77375. doi: 10.1002/advs.77375 (PMC13502350; doi:10.1002/advs.77375)
Supplement: Supplementary file 1 [file ADVS-13-e77375-s001.docx]

Supporting Information

**A Reconfigurable Memristive Spiking Neuron Enabling Advanced Neuromorphic Computing**

Pek Jun Tiw, Yuqi Li, Zhongyuan Li, Qihang Ding, Yuzhe Wang, Jiarong Wang, and Yuchao Yang^*^

**Figure S1.** Simulated evolution of *V*_ctrl1_, *V*_ctrl2_, *V*_mem_ and the spike output of the neuron in the (a) fast-spiking, (b) adaptive spiking, (c) phasic bursting, and (d) single-spiking regimes when subjected to a periodic *I*_in_ pulse train. The *I*_in_ pulse train has an amplitude of 1.1 mA, a pulse period of 2 μs and a duty cycle of 50%. These responses are similar to those obtained under constant input stimuli.

**Figure S2.** Simulated evolution of *V*_ctrl1_, *V*_ctrl2_, *V*_mem_ and the spike output of the neuron in the (a) fast-spiking, (b) adaptive spiking, (c) phasic bursting, and (d) single-spiking regimes when subjected to the same *I*_in_ pulse train, in which the amplitude and duration of every *I*_in_ pulse as well as the intervals between consecutive pulses were randomly generated. In the fast-spiking regime, the neuron responds quickly to high-frequency large amplitude pulses effectively. In the adaptive spiking regime, the neuron mainly responds to strong input pulses that are spaced sufficiently apart. The neuron responds to strong input pulses in the early phase of the entire input duration with multiple spikes and only one spike when biased in the phasic bursting regime and the single-spiking regime, respectively.

**Figure** **S3.** Shifting of the *V*_g1_ boundary between adaptive spiking and fast-spiking under different *V*_th,VO2_. The boundary shifts towards lower *V*_g1_ values when *V*_th,VO2_ increases from (a) 2.7 V, (b) 2.9 V, (c) 3.1 V to (d) 3.3 V.

**Figure** **S4.** Shifting of the *V*_g2_ boundary between adaptive spiking and fast-spiking under different *V*_th,VO2_. The boundary shifts towards lower *V*_g2_ values when *V*_th,VO2_ increases from (a) 2.7 V, (b) 2.9 V, (c) 3.1 V to (d) 3.3 V.

**Figure S5.** Experimentally measured *V*_ctrl2_, *V*_mem_, and spike output in the fast-spiking regime when *V*_in_ is varied. The spiking frequency (*f*_sp_) increases with increasing *V*_in_. *V*_g1_ and *V*_g2_ are fixed at 5 V and 6 V, respectively, for all measurements.

**Figure S6.** Experimentally measured *V*_ctrl2_, *V*_mem_, and spike output in the single-spiking regime when *V*_in_ is varied. The time to spike (*t*_sp_) decreases with increasing *V*_in_. *V*_g1_ and *V*_g2_ are both fixed at 10 V for all measurements.

**Figure S7.** Experimentally measured *V*_ctrl2_, *V*_mem_, and spike output in the phasic bursting regime when *V*_in_ is varied. The period between spikes within a burst (*T*_burst_) decreases with increasing *V*_in_. *V*_g1_ and *V*_g2_ are fixed at 10 V and 6 V, respectively, for all measurements.

**Figure S8.** Experimentally measured *V*_ctrl2_, *V*_mem_, and spike output in the phasic bursting regime when *V*_g2_ is varied. The number of spikes within a burst (*N*_sp_) decreases with increasing *V*_g2_. *V*_in_ and *V*_g1_ are both fixed at 10 V for all measurements.

**Figure S9.** Experimentally measured *V*_ctrl2_, *V*_mem_, and spike output in the adaptive spiking regime when *V*_in_ is varied. The initial spiking period (*T*_ini_) decreases with increasing *V*_in_. *V*_g1_ and *V*_g2_ are fixed at 8 V and 6 V, respectively, for all measurements.

**Figure S10.** Experimentally measured *V*_ctrl2_, *V*_mem_, and spike output in the adaptive spiking regime when *V*_g2_ is varied. The onset of adaptation, defined by the number of spikes before the spiking period increases (*N*_o_), decreases with increasing *V*_g2_. *V*_in_ and *V*_g1_ are fixed at 10 V and 8.2 V, respectively, for all measurements.

**Figure S11.** Experimentally measured *V*_ctrl2_, *V*_mem_, and spike output in the adaptive spiking regime when *V*_g1_ is varied. The post-adaptation saturated spiking period (*T*_sat_) increases with increasing *V*_g1_. *V*_in_ and *V*_g2_ are fixed at 10 V and 6 V, respectively, for all measurements.

**Figure** **S12.** Variations in spike train parameters under *V*_g1,2_ noise. (a) *C*_v,_*_N_*_o_, (b) *C*_v,_*_T_*_ini_ and (c) *C*_v,_*_T_*_sat_ versus *V*_g1_ under different levels of *V*_g1_ noise when *V*_g1_ is varied in the adaptive spiking regime. (d) *C*_v,_*_N_*_o_, (e) *C*_v,_*_T_*_ini_ and (f) *C*_v,_*_T_*_sat_ versus *V*_g2_ under different levels of *V*_g2_ noise when *V*_g2_ is varied in the adaptive spiking regime. (g) *C*_v,_*_N_*_sp_ and (h) *C*_v,_*_T_*_burst_ versus *V*_g2_ under different levels of *V*_g2_ noise when *V*_g2_ is varied in the phasic bursting regime.

**Figure** **S13.** Variations in spike train parameters under *V*_dd_ noise. (a) *C*_v,_*_N_*_o_, (b) *C*_v,_*_T_*_ini_ and (c) *C*_v,_*_T_*_sat_ versus *V*_g1_ under different levels of *V*_dd_ noise when *V*_g1_ is varied in the adaptive spiking regime. (d) *C*_v,_*_N_*_o_, (e) *C*_v,_*_T_*_ini_ and (f) *C*_v,_*_T_*_sat_ versus *V*_g2_ under different levels of *V*_dd_ noise when *V*_g2_ is varied in the adaptive spiking regime. (g) *C*_v,_*_N_*_sp_ and (h) *C*_v,_*_T_*_burst_ versus *V*_g2_ under different levels of *V*_dd_ noise when *V*_g2_ is varied in the phasic bursting regime.

**Figure** **S14.** Variations in spike train parameters under *V*_th,VO2_ variations. (a) *C*_v,_*_N_*_o_, (b) *C*_v,_*_T_*_ini_ and (c) *C*_v,_*_T_*_sat_ versus *V*_g1_ under different levels of *V*_th,VO2_ variations when *V*_g1_ is varied in the adaptive spiking regime. (d) *C*_v,_*_N_*_o_, (e) *C*_v,_*_T_*_ini_ and (f) *C*_v,_*_T_*_sat_ versus *V*_g2_ under different levels of *V*_th,VO2_ variations when *V*_g2_ is varied in the adaptive spiking regime. (g) *C*_v,_*_N_*_sp_ and (h) *C*_v,_*_T_*_burst_ versus *V*_g2_ under different levels of *V*_th,VO2_ variations when *V*_g2_ is varied in the phasic bursting regime.

**Figure** **S15.** Variations in spike train parameters under CMOS process variations. (a) *C*_v,_*_N_*_o_, (b) *C*_v,_*_T_*_ini_ and (c) *C*_v,_*_T_*_sat_ versus *V*_g1_ under CMOS process variations when *V*_g1_ is varied in the adaptive spiking regime. (d) *C*_v,_*_N_*_o_, (e) *C*_v,_*_T_*_ini_ and (f) *C*_v,_*_T_*_sat_ versus *V*_g2_ under CMOS process variations when *V*_g2_ is varied in the adaptive spiking regime. (g) *C*_v,_*_N_*_sp_ and (h) *C*_v,_*_T_*_burst_ versus *V*_g2_ under CMOS process variations when *V*_g2_ is varied in the phasic bursting regime.

**Figure** **S16.** Network performance in the hazard avoidance task under various hardware nonidealities. (a) Mean test accuracy and (b) Mean Squared Relative Errors (MSRE) of spike train parameters under varying levels of *V*_th,VO2_ variations. (c) Mean test accuracy and (d) MSRE of spike train parameters under varying levels of MOSFET threshold voltage variations. (e) Mean test accuracy and (f) MSRE of spike train parameters under varying levels of *V*_g1,2_ noise. (g) Mean test accuracy and (h) MSRE of spike train parameters under varying levels of *V*_dd_ noise.

**Figure S17.** Additional network comparisons on the EPHNOGRAM and SHD datasets. (a) Comparison of the maximum test accuracies between heterogeneous LSNNs with and without *V*_g1_ enabled, each averaged over *n* = 6 randomly initialized trials. Error bars represent ±1 standard deviation from the mean. No significant difference in test accuracy is observed. (b) Comparison of the maximum test accuracies between three LSNNs, each averaged over *n* = 6 randomly initialized trials. Error bars represent ±1 standard deviation from the mean. The sizes of the smaller homogeneous LSNNs and the dynamic LSNNs are the same, 5×200×5 for the EPHNOGRAM task and 351×200×20 for the SHD task. The sizes of the larger heterogeneous LSNNs are 5×284×5 for the EPHNOGRAM task and 351×324×20 for the SHD task. This ensures that the total number of trainable parameters in the larger heterogeneous LSNNs (83496 for EPHNOGRAM and 225180 for SHD) is roughly equal to that of the dynamic LSNNs (83000 for EPHNOGRAM and 224400 for SHD). The larger heterogeneous LSNN cannot match the performance of the dynamic LSNN, indicating that the dynamic LSNN's improved performance cannot be attributed solely to its larger number of weights. Instead, the network-coupled neuron dynamics, or on-the-fly neuromodulations, could be the factor contributing to the increased temporal computing capability.

**Figure** **S18.** A comparison of the adaptation index (AI) between the neuron population observed in the mouse visual cortex^[1]^ and the hidden neurons in the heterogeneous LSNN with trainable *V*_g1_. The adaptation index quantifies the rate of change of the inter-spike intervals (ISI) of a spike train in response to an input step stimulus. AI is defined as follows:

$$\mathrm{AI}=\frac{1}{N-1}\sum_{n=1}^{N-1} \frac{\mathrm{ISI}_{n+1}-\mathrm{ISI}_{n}}{\mathrm{ISI}_{n+1}+\mathrm{ISI}_{n}}$$

AI > 0 indicates the presence of spike frequency adaptation. ISI*_n_* is the interval between the *n*-th and (*n*+1)-th spike in the spike train, and *N* is the total number of spikes. The distribution of AI of adaptive neurons in the heterogeneous LSNN agrees well with that of biological neurons in the cortex.

**Figure S19.** Additional examples showing the evolution of *V*_ctrl2_ of hidden neurons in a dynamic LSNN when presented with two different input samples. The five panels in (a) and (b) in the same relative positional order correspond to the same neuron in the same training trial. In addition to exhibiting negative imprinting, each neuron tends to respond to a different combination of features. For example, the neuron in the bottom-most panels (corresponding to the same neuron in the bottom-most panel of Figure 5h in the main text) tends to respond to features just before the most prominent peak in the ECG signal, regardless of how many peaks are present in a sample. The neuron in the fourth panel, however, elicits a response after each of the two most prominent features in the ECG signal ends.

**Figure** **S20.** Mean test accuracy of the heterogeneous LSNN in the EPHNOGRAM task under varying levels of (a) *V*_th,VO2_ variations, (b) MOSFET threshold voltage variations, (c) *V*_g1,2_ noise, and (d) *V*_dd_ noise.

**Figure** **S21.** Mean test accuracy of the dynamic LSNN in the EPHNOGRAM task under varying levels of (a) *V*_th,VO2_ variations, (b) MOSFET threshold voltage variations, (c) *V*_g1,2_ noise, and (d) *V*_dd_ noise.

**Figure S22.** Experimental setup for characterizing the multi-mode reconfigurable neuron.

**Supporting Table 1: SPICE simulation circuit parameters**

| **Device** | **Parameters** |
| --- | --- |
| *M*_1_ | pmos_6p0: *L* = 5 μm, *W* = 0.3 μm |
| *M*_2_ | pmos_6p0: *L* = 2 μm, *W* = 0.3 μm |
| *M*_3_ | nmos_6p0: *L* = 0.6 μm, *W* = 0.3 μm |
| *M*_4_ | nmos_6p0: *L* = 0.6 μm, *W* = 0.75 μm, *m* = 6 |
| *R*_1_ | ppolyf_u_1k_6p0: *L* = 0.28 μm, *W* = 2 μm |
| *C*_1_ | mim_2p0fF: *L* = 120 μm, *W* = 125 μm |
| *C*_2_ | mim_2p0fF: *L* = 20 μm, *W* = 25 μm |
| *C*_3_ | Parasitic: 1nF |
| VO_2_ | *V*_th,VO2_ ≈ 3 V, *V*_hold,VO2_ ≈ 0.6 V |

**Supporting Table 2: Energy and power consumption of the reconfigurable neuron**

| **Regime** | ***N*** | ***E*_int_ (nJ)** | ***E*_ctrl_ (pJ)** | ***E*_sp_ (nJ)** | ***P*_avg_ (mW)** | **Bias** |
| --- | --- | --- | --- | --- | --- | --- |
| Single-spiking | 1 | 6.001 | 36.65 | 6.038 | 0.7700 | *V*_g1_ = 0 V, *V*_g2_ = 6 V |
| Phasic bursting | 10 | 69.59 | 268.7 | 6.986 | 0.9204 | *V*_g1_ = 6 V, *V*_g2_ = 3 V |
| Adaptive spiking | 27 | 1051 | 667.2 | 38.96 | 1.087 | *V*_g1_ = 4.8 V, *V*_g2_ = 3 V |
| Fast-spiking | 170 | 1002 | 5607 | 5.930 | 1.005 | *V*_g1_ = 0 V, *V*_g2_ = 0 V |

**Supporting Table 3: Fitted circuit parameters for simulating the tunability of spike train parameters**

| **Parameter** | **Value** | **Units** |
| --- | --- | --- |
| Memristor parameters | | |
| *V*_th,VO2_ | 5.1 | V |
| *V*_hold,VO2_ | 0.6 | V |
| *R*_VO2_ (*R*_1_ included) | 24 | kΩ |
| *C*_3_ | 0.47 | nF |
| MOSFET parameters | | |
| *\|V*_th,_*_M_*_1_\| | 0.9 | V |
| *K_M_*_1_ | 0.32 | mA V^-2^ |
| \|*V*_th,_*_M_*_2_\| | 2.6 | V |
| *K_M_*_2_ | 5 | mA V^-2^ |
| *V*_th,_*_M_*_4_ | 0.7 | V |
| *K_M_*_4_ | 0.64 | mA V^-2^ |
| Other circuit parameters | | |
| *C*_1_ | 10 | nF |
| *C*_2_ | 1.05 | nF |
| Δ*V*_sp_ | 2.2 | V |
| *R*_2_ | 11 | kΩ |
| *V*_dd_ | 10 | V |

**Supporting Table 4: Comparison with existing works**

|  | **Ref**^[2]^ | **Ref**^[3]^ | **Ref**^[4]^ | **This work** |
| --- | --- | --- | --- | --- |
| Neuron model | HH | Modified LIF | LIF | Modified LIF |
| Multi-mode spiking | Yes | Yes | No | **Yes** |
| Reconfigurability | Yes | Yes | No | **Yes** |
| Freely tunable parameters (for spiking modes with > 2 parameters) | No | No | No | **Yes** |
| **Multiplexed coding** | | | | |
| Regimes per channel demonstrated | 2 | 1 | 1 | **3** |
| Maximum multiplexed coding states per regime demonstrated | 1 | 1 | 2 | **3** |
| Total multiplexed coding states per channel demonstrated | 2 | 1 | 2 | **6** |
| **SNNs with complexity** | | | | |
| Demonstrated SNNs with complexity | No | No | No | **Yes** |
| Support for heterogeneity^*^ | No | Possible | No | **Yes** |
| Support for dynamic mode switching^†^ | Limited | Limited | No | **Yes** |

***** Heterogeneity in terms of intra-mode parameters (e.g., adaptation time constant) or the presence of different spiking modes is considered, all of which should be freely achieved without changing circuit elements.

**†** Dynamic mode switching requires functional-specific ports (akin to neuromodulatory projections in the brain) to be biologically plausible enough to support a wider range of networks.

**Supporting Table 5: Network parameters**

| **Parameter** | **Value** | **Units** |
| --- | --- | --- |
| Reconfigurable neuron parameters | | |
| *V*_th,VO2_ | 5.1 | V |
| *V*_hold,VO2_ | 0.6 | V |
| *R*_VO2_ (*R*_1_ included) | 24 | kΩ |
| *C*_3_ | 0.83 | nF |
| \|*V*_th,_*_M_*_1_\| | 0.9 | V |
| *K_M_*_1_ | 0.32 | mA V^-2^ |
| \|*V*_th,_*_M_*_2_\| | 2.6 | V |
| *K_M_*_2_ | 5 | mA V^-2^ |
| *V*_th,_*_M_*_4_ | 0.7 | V |
| *K_M_*_4_ | 0.64 | mA V^-2^ |
| *C*_1_ | 10 | nF |
| *C*_2_ | 0.3 | nF |
| Δ*V*_sp_ | 7 | V |
| *V*_dd_ | 10 | V |
| Other network parameters | | |
| Δ*t* | 1 | μs |
| *τ*_low-pass_ | 20 | Δ*t* |
|  |  |  |

**Supporting Note 1: Derivation of regime boundaries**

This section presents the derivations of all spike train parameters and regime boundaries. For convenience, we list out the equation numbers referring to the final equations describing these parameters and boundaries as follows:

Spike train parameters:

- *T*_burst_: Equations 5-7
- *T*_ini_: Equations 5-7
- *f*_sp_: Equations 5-8
- *t*_sp_: Equation 9
- *T*_sat_: Equation 11, Equations 15-16
- *N*_sp_: Equation 21
- *N*_o_: Equation 24

Regime boundaries:

- Fast-spiking and adaptive spiking: Equations 25-26
- Adaptive spiking and phasic bursting: Equation 27
- Single-spiking: Equation 30-31

Spike train parameters

First, we consider the intrinsic spiking period (*T*_burst_ during burst coding, *T*_ini_ during adaptive rate coding) or the frequency (*f*_sp_ during normal rate coding) of the integrator module. This can be derived by first solving the dynamical equation at node *V*_mem_, given by Equations 1 (*V*_in_-driven) or 2 (*I*_in_-driven).

$$\begin{aligned} C_{3}\frac{d}{dt}V_{\mathrm{mem}}=\frac{V_{\mathrm{in}}}{R_{2}}-\frac{V_{\mathrm{mem}}}{R_{VO2}}\#\left( 1 \right) \end{aligned}$$

$$\begin{aligned} C_{3}\frac{d}{dt}V_{\mathrm{mem}}=I_{\mathrm{in}}-\frac{V_{\mathrm{mem}}}{R_{VO2}}\#\left( 2 \right) \end{aligned}$$

Note that *R*_VO2_ here includes the series resistor *R*_1_ in the discharging path. In the *V*_in_-driven case, we obtain Equation 3 with the boundary condition *V*_mem_(0) = *V*_0_.

$$\begin{aligned} V_{\mathrm{mem}}\left( t \right)=\beta V_{\mathrm{in}}-\left( \beta V_{\mathrm{in}}-V_{0} \right)\exp\left( -\frac{t}{\tau_{m}} \right)\#\left( 3 \right) \end{aligned}$$

*τ*_m_ = (*R*_VO2_||*R*_2_)∙*C*_3_ is the membrane time constant, *β* = *R*_VO2_/(*R*_2_+*R*_VO2_) is the voltage divider term for node *V*_mem_. Rearranging, we have Equation 4.

$$\begin{aligned} t=\tau_{m}\ln\left( \frac{\beta V_{\mathrm{in}}-V_{0}}{\beta V_{\mathrm{in}}-V_{\mathrm{mem}}\left( t \right)} \right)\#\left( 4 \right) \end{aligned}$$

The rise and fall times (*t*_r_ and *t*_f_) between *V*_hold,VO2_ and *V*_th,VO2_ can be obtained (Equations 5-6).

$$\begin{aligned} t_{r}=\tau_{m,off}\cdot\ln\left( \frac{\beta_{\mathrm{off}}V_{\mathrm{in}}-V_{hold,VO2}}{\beta_{\mathrm{off}}V_{\mathrm{in}}-V_{th,VO2}} \right)\#\left( 5 \right) \end{aligned}$$

$$\begin{aligned} t_{f}=\tau_{m,on}\cdot\ln\left( \frac{V_{th,VO2}-\beta_{\mathrm{on}}V_{\mathrm{in}}}{V_{hold,VO2}-\beta_{\mathrm{on}}V_{\mathrm{in}}} \right)\#\left( 6 \right) \end{aligned}$$

where the subscript “on” or “off” means substituting *R*_VO2_ with *R*_VO2,on_ or *R*_VO2,off_. The spiking period and frequency are thus

$$\begin{aligned} T_{\mathrm{burst}}=T_{\mathrm{ini}}=t_{r}+t_{f}\#\left( 7 \right) \end{aligned}$$

$$\begin{aligned} f_{\mathrm{sp}}=\frac{1}{T}\#\left( 8 \right) \end{aligned}$$

In the *I*_in_-driven case, *τ*_m_ = *R*_VO2_*C*_3_ and *βV*_in_ should be substituted with *I*_in_*R*_VO2_. Therefore, *T*_burst_ during burst coding and *T*_ini_ during adaptive rate coding are both given by Equation 7, whereas *f*_sp_ during normal rate coding is given by Equation 8. During single-spike coding, *t*_sp_ is simply the rise time from 0 V to *V*_th,VO2_ given by Equation 9 (or its *I*_in_-driven equivalent).

$$\begin{aligned} t_{\mathrm{sp}}=\tau_{m,off}\cdot\ln\left( \frac{\beta_{\mathrm{off}}V_{\mathrm{in}}}{\beta_{\mathrm{off}}V_{\mathrm{in}}-V_{th,VO2}} \right)\#\left( 9 \right) \end{aligned}$$

Next, we consider the post-adaptation saturated spiking period (*T*_sat_ during adaptive rate coding). This can be split into multiple cases depending on the operating regime of *M*_1_ and *M*_2_. During adaptation, *V*_ctrl2_ is in an equilibrium state in which it oscillates between two values separated by Δ*V*_sp_. *T*_sat_ is therefore the time to charge *C*_2_ by Δ*V*_sp_. Using Kirchhoff’s current law at node *V*_ctrl1_, we have Equation 10.

$$\begin{aligned} \left| I_{ds,M1} \right|=C_{1}\frac{\Delta V_{ctrl1}}{\Delta t}+\left| I_{ds,M2} \right|=C_{1}\frac{\Delta V_{ctrl1}}{\Delta t}+C_{2}\frac{\Delta V_{ctrl2}}{\Delta t}\#\left( 10 \right) \end{aligned}$$

We first consider the case where *M*_1_ is saturated (*V*_g1_ is relatively high). If *V*_g2_ is also relatively high, such that *M*_2_ is always in saturation, |*I*_ds,_*_M_*_2_| is small, and *V*_ctrl1_ exhibits negligible fluctuations. Thus, Δ*V*_ctrl1_/Δ*t* ≈ 0. Substituting Δ*V*_ctrl2_ with Δ*V*_sp_ and Δ*t* with *T*_sat_ and rearranging, we have Equation 11.

$$\begin{aligned} T_{\mathrm{sat}}=\frac{C_{2}\Delta V_{\mathrm{sp}}}{\left| I_{ds,M1,sat} \right|}=\frac{C_{2}\Delta V_{\mathrm{sp}}}{\frac{1}{2}K_{M1}\left( V_{\mathrm{dd}}-V_{g1}-\left| V_{th,M1} \right| \right)^{2}}\#\left( 11 \right) \end{aligned}$$

If *V*_g2_ is not high enough, *M*_2_ operates first in the saturation regime and then in the linear regime within a spiking period. In this case, before each spike, *V*_ctrl1_ = *V*_ctrl2_ = *V*_pre_. After each spike, *V*_ctrl2_ = *V*_pre_-Δ*V*_sp_. *T*_sat_ can be divided into two parts. *t*_a_ represents the first part where *M*_2_ is in saturation, *V*_ctrl1_ decreases from *V*_pre_ to *V*_A_, and *V*_ctrl2_ increases from *V*_pre_-Δ*V*_sp_ to *V*_A_, where *V*_A_ is the value of *V*_ctrl1_ and *V*_ctrl2_ after the charge-sharing process. *t*_b_ is the second part where *M*_2_ is in the linear regime, *V*_ctrl1_ and *V*_ctrl2_ increase together from *V*_A_ to *V*_pre_. Substituting relevant values for the durations *t*_a_ and *t*_b_ into Equation 10 and rearranging, we have Equations 12-13.

$$\begin{aligned} t_{a}=\frac{{C_{1}\left( V_{A}-V_{\mathrm{pre}} \right)+C}_{2}\left( V_{A}-V_{\mathrm{pre}}+\Delta V_{\mathrm{sp}} \right)}{\left| I_{ds,M1,sat} \right|}\#\left( 12 \right) \end{aligned}$$

$$\begin{aligned} t_{b}=\frac{\left( C_{1}+C_{2} \right)\left( V_{\mathrm{pre}}-V_{A} \right)}{\left| I_{ds,M1,sat} \right|}\#\left( 13 \right) \end{aligned}$$

Thus, upon summing, *T*_sat_ is simply given by Equation 14, which is conveniently equal to Equation 11.

$$\begin{aligned} T_{\mathrm{sat}}=\frac{C_{2}\Delta V_{\mathrm{sp}}}{\left| I_{ds,M1,sat} \right|}\#\left( 14 \right) \end{aligned}$$

Now, we consider the case where *M*_1_ is in the linear regime (*V*_g1_ is relatively low). If *V*_g2_ is relatively high, such that *M*_2_ is always in saturation, |*I*_ds,_*_M_*_2_| is small, and *V*_ctrl1_ exhibits negligible fluctuations. Thus, Δ*V*_ctrl1_/Δ*t* ≈ 0. Substituting Δ*V*_ctrl2_ with Δ*V*_sp_ and Δ*t* with *T*_sat_ in Equation 10 and rearranging, we have Equation 15.

$$\begin{aligned} T_{\mathrm{sat}}=\frac{C_{2}\Delta V_{\mathrm{sp}}}{\left| I_{ds,M1,lin} \right|}=\frac{C_{2}\Delta V_{\mathrm{sp}}}{\frac{1}{2}K_{M1}\left[ 2\left( V_{\mathrm{dd}}-V_{g1}-\left| V_{th,M1} \right| \right)-\left( V_{\mathrm{dd}}-V_{ctrl1} \right) \right]\left( V_{\mathrm{dd}}-V_{ctrl1} \right)}\#\left( 15 \right) \end{aligned}$$

*V*_ctrl1_ can be eliminated from Equation 15 via the relation in Equation 16.

$$\begin{aligned} \left| I_{ds,M1,lin} \right|=\left| I_{ds,M2,sat} \right|\#\left( 16 \right) \end{aligned}$$

If *V*_g2_ is further reduced, the neuron is most likely in the fast-spiking regime. Note that the drain-source current through *M*_1_ and *M*_2_ can be large if |*V*_ds_| > 0. Even if *T*_ini_ is sufficiently small such that the inequality *T*_sat_ > *T*_ini_ still holds, *T*_sat_ is so small that using it to represent information or perform computations becomes arguably impractical.

Finally, we consider when the control module begins to significantly disrupt the intrinsic dynamics of the integrator (*N*_sp_ during burst coding, *N*_o_ during adaptive rate coding). First, we consider the case of bursting. We define the magnitude of *V*_ctrl2_ needed for *V*_mem_ to reach *V*_th,VO2_ asymptotically as *V*_ctrl2,th_. In this scenario, d*V*_mem_/d*t* approaches zero. *M*_4_ is in saturation as *V*_gs_ is only very slightly larger than *V*_th_. Using Kirchhoff’s current law at node *V*_mem_, expanding *I*_ds,_*_M_*_4_, and rearranging, we have Equation 17.

$$\begin{aligned} V_{ctrl2,th}=V_{th,VO2}+V_{th,M4}+\sqrt{\frac{2V_{th,VO2}}{R_{VO2}K_{M4}}}\#\left( 17 \right) \end{aligned}$$

For a given *V*_g2_, the minimum achievable *V*_ctrl1_ is *V*_g2_+|*V*_th,_*_M_*_2_|. This is the threshold needed to turn *M*_2_ on to reduce *V*_ctrl1_. Now, we approach the main derivation from the conservation of charge perspective. Initially, *V*_ctrl1_ = *V*_ctrl2_ = *V*_dd_. After firing a burst of spikes, *V*_ctrl1_ approaches *V*_g2_+|*V*_th,_*_M_*_2_|, while *V*_ctrl2_ is at some value *V*_B_ < *V*_ctrl2,th_. The total difference in charge stored in *C*_1_ and *C*_2_ is thus given by Equation 18.

$$\begin{aligned} \Delta Q_{c}=C_{1}\left[ \left( V_{g2}+\left| V_{th,M2} \right| \right)-V_{\mathrm{dd}} \right]+C_{2}\left( V_{B}-V_{\mathrm{dd}} \right)\#\left( 18 \right) \end{aligned}$$

This difference in charge is caused by *M*_3_ discharging the capacitors in response to each spike. Thus, we have Equation 19.

$$\begin{aligned} \Delta Q_{\mathrm{sp}}=-N_{\mathrm{sp}}C_{2}\Delta V_{\mathrm{sp}}\#\left( 19 \right) \end{aligned}$$

Equating Equations 18-19 and rearranging, we have Equation 20.

$$\begin{aligned} N_{\mathrm{sp}}=\frac{1}{\Delta V_{\mathrm{sp}}}\left[ \frac{C_{1}}{C_{2}}\left( V_{\mathrm{dd}}-V_{g2}-\left| V_{th,M2} \right| \right)+V_{\mathrm{dd}}-V_{B} \right]\#\left( 20 \right) \end{aligned}$$

To arrive at the final form (Equation 21), *V*_B_ can be replaced by *V*_ctrl2,th_, and the resulting expression should be evaluated using the ceiling function.

$$\begin{aligned} N_{\mathrm{sp}}=\left\lceil\frac{1}{\Delta V_{\mathrm{sp}}}\left[ \frac{C_{1}}{C_{2}}\left( V_{\mathrm{dd}}-V_{g2}-\left| V_{th,M2} \right| \right)+V_{\mathrm{dd}}-V_{ctrl2,th} \right] \right\rceil\#\left( 21 \right) \end{aligned}$$

Now, we consider the case of adaptive spiking. This regime differs from bursting in that *M*_1_ is conducting and supplying charges to the *C*_1_-*C*_2_ network. The total charge injected into the capacitors up until the onset of adaptation is given by Equation 22.

$$\begin{aligned} \Delta Q_{\mathrm{inj}}=N_{o}T_{\mathrm{ini}}\left| I_{ds,M1} \right|\#\left( 22 \right) \end{aligned}$$

Thus, the difference in charge in the *C*_1_-*C*_2_ network is the net result of charge injection by *M*_1_ and charge removal by *M*_3_ (Equation 23).

$$\begin{aligned} \Delta Q_{c}=\Delta Q_{\mathrm{inj}}+\Delta Q_{\mathrm{sp}}\#\left( 23 \right) \end{aligned}$$

After substitution, rearranging, replacing *V*_B_, and evaluating the ceiling of the resulting expression, we have the final form in Equation 24.

$$\begin{aligned} N_{o}=\left\lceil\frac{C_{1}\left( V_{\mathrm{dd}}-V_{g2}-\left| V_{th,M2} \right| \right)+C_{2}\left( V_{\mathrm{dd}}-V_{ctrl2,th} \right)}{C_{2}\Delta V_{\mathrm{sp}}-T_{\mathrm{ini}}\left| I_{ds,M1} \right|} \right\rceil\#\left( 24 \right) \end{aligned}$$

Regime boundaries

We can define the boundary between fast-spiking and adaptive spiking using *T*_sat_ = *T*_ini_. This is consistent with the definition of the adaptation index, AI, given in Figure S18, in which adaptation corresponds to AI > 0, and therefore *T*_sat_ > *T*_ini_. Since *T*_sat_ has two definitions depending on the operating regime of *M*_1_ and *M*_2_, this boundary can be divided into two parts. If *M*_1_ is in the saturation regime, the boundary ((i) in Figure 2g of the main text, Equation 25) is given by equating Equations 7 and 11.

$$\begin{aligned} V_{g1}=V_{\mathrm{dd}}-\left| V_{th,M1} \right|-\sqrt{\frac{2C_{2}\Delta V_{\mathrm{sp}}}{\left( t_{r}+t_{f} \right)K_{M1}}}\#\left( 25 \right) \end{aligned}$$

If *M*_1_ is in the linear regime, the boundary ((ii) in Figure 2g of the main text, Equation 26) is given by equating Equations 7 and 15-16 (elimination of *V*_ctrl1_).

$$\begin{aligned} V_{ctrl1+}\left( V_{g1}, V_{g2} \right)=\sqrt{\frac{2C_{2}\Delta V_{\mathrm{sp}}}{\left( t_{r}+t_{f} \right)K_{M2}}}\#\left( 26 \right) \end{aligned}$$

This boundary is expressed implicitly in terms of *V*_g1_ and *V*_g2_. *V*_ctrl1+_ is the positive root of the quadratic equation in Equation 16.

The neuron is in the phasic bursting regime if there is no pathway for injecting charge into the *C*_1_-*C*_2_ network. This corresponds to when *M*_1_ is turned off. Therefore, the boundary is given by the threshold of *M*_1_ (Equation 27).

$$\begin{aligned} V_{g1}=V_{\mathrm{dd}}-\left| V_{th,M1} \right|\#\left( 27 \right) \end{aligned}$$

In the single-spiking regime, *V*_ctrl2_ needs to be maintained below *V*_ctrl2,th_ after only one spike. We first consider the case in which *M*_1_ is turned off. We again use the conservation of charge perspective. After firing one spike, *V*_ctrl2_ must decrease from *V*_dd_ to a value below *V*_ctrl2,th_ and simultaneously *V*_ctrl1_ must decrease from *V*_dd_ to *V*_g2_+|*V*_th,_*_M_*_2_| to turn *M*_2_ off to prevent further charging of *C*_2_. Thus, at the boundary, we have the following changes in the amount of stored charge (Equations 28-29).

$$\begin{aligned} \Delta Q_{c}=C_{1}\left[ \left( V_{g2}+\left| V_{th,M2} \right| \right)-V_{\mathrm{dd}} \right]+C_{2}\left( V_{ctrl2,th}-V_{\mathrm{dd}} \right)\#\left( 28 \right) \end{aligned}$$

$$\begin{aligned} \Delta Q_{\mathrm{sp}}=-C_{2}\Delta V_{\mathrm{sp}}\#\left( 29 \right) \end{aligned}$$

Equating these two expressions and rearranging, we have Equation 30.

$$\begin{aligned} V_{g2}=V_{\mathrm{dd}}-\left| V_{th,M2} \right|-\frac{C_{2}}{C_{1}}\left( V_{ctrl2,th}+\Delta V_{\mathrm{sp}}-V_{\mathrm{dd}} \right)\#\left( 30 \right) \end{aligned}$$

If *M*_1_ is turned on, node *V*_ctrl1_ receives a sustained charge injection and tends towards *V*_dd_. Thus, if *M*_2_ is initially biased to turn on even slightly, it will remain on and inject charge into *C*_2_, allowing the circuit to fire persistently. Therefore, *M*_2_ must be off initially, and the boundary is now given by Equation 31.

$$\begin{aligned} V_{g2}=V_{\mathrm{dd}}-\left| V_{th,M2} \right|\#\left( 31 \right) \end{aligned}$$

In practise, *C*_2_ is much smaller than *C*_1_ to ensure proper operation of the neuron, which depends on the gradual decrease of *V*_ctrl1_ from *V*_dd_ and the oscillatory-like dynamics of *V*_ctrl2_. Thus, the difference between Equation 30 and 31 is small. Besides, even if *M*_1_ is turned on while *V*_g2_ is biased between Equations 30 and 31, *T*_sat_ is so large (Figure 3h in the main text) that in the typical timescale of a task, the neuron effectively fires only one spike (Figure 2g in the main text). Therefore, to prevent clutter in the main text, we give Equation 30 as the only boundary.

**Supporting Note 2: Algorithmic approach for obtaining the phase diagrams**

In Figure 2h, boundaries (iii) and (iv) can be obtained directly by determining if *V*_g1_ or *V*_g2_ meets the threshold described by Equations 3 and 4 in the main text. Boundary (i) can also be solved easily from Equation 1 in the main text. Boundary (ii), however, is not easily solvable by hand. There are two ways to obtain this boundary. One way is to calculate it using symbolic math. The SymPy module was used for this purpose to obtain the solid boundary (ii) line plotted in Figure 2h. The other way is to simply simulate the dynamics of the neuron around this boundary and determine from the ISIs in the output spike train if adaptation exists. The coloring of the spiking regimes in the phase diagrams was obtained using this method. We summarize the algorithmic approach used to obtain the boundaries and to determine the spiking mode given *V*_g1_, *V*_g2_ and *V*_in_ below:

**procedure** determine_mode(*V*_g1_, *V*_g2_, *V*_in_)

Calculate *T*_ini_, *V*_ctrl2,th_

*V*_g1,boundary1_ = *V*_dd_ – |*V*_th,_*_M_*_1_| - sqrt(2 * *C*_2_ * Δ*V*_sp_ / (|*K_M_*_1_| * *T*_ini_))	// Boundary (i)

*V*_g1,boundary3_ = *V*_dd_ – |*V*_th,_*_M_*_1_|	// Boundary (iii)

*V*_g2,boundary4_ = *V*_dd_ – |*V*_th,_*_M_*_2_| - *C*_2_ / *C*_1_ * (*V*_ctrl2,th_ + Δ*V*_sp_ - *V*_dd_)	// Boundary (iv)

**if** *V*_g2_ ≥ *V*_g2,boundary4_, **then**

Mode is single-spiking

**else if** *V*_g1_ ≥ *V*_g1,boundary3_, **then**

Mode is phasic bursting

**else**

**if** use symbolic math, **then**

LHS = |*I*_ds,_*_M_*_2,sat_(*V*_ctrl1,symbol_, *V*_g2,symbol_)|

RHS = |*I*_ds,_*_M_*_1,lin_(*V*_ctrl1,symbol_, *V*_g1,symbol_)|

// Solve for *V*_ctrl1,symbol_ in terms of *V*_g1,symbol_ and *V*_g2,symbol_, use only the positive root (*V*_ctrl1,symbol_^+^)

*V*_ctrl1,symbol_^+^, *V*_ctrl1,symbol_^–^ = solve(equality(LHS, RHS), *V*_ctrl1,symbol_)

LHS = *V*_ctrl1,symbol_^+^- *V*_g2,symbol_

RHS = |*V*_th,_*_M_*_2_| + sqrt(2 * *C*_2_ * Δ*V*_sp_ / (|*K_M_*_1_| * *T*_ini_))

// Solve for *V*_g2,symbol_ in terms of *V*_g1,symbol_, use only the positive root (*V*_g2,symbol_^+^)

*V*_g2,symbol_^+^, *V*_g2,symbol_^–^ = solve(equality(LHS, RHS), *V*_g2,symbol_)

**if** *V*_g1_ > *V*_g1,boundary1_, **then**

Mode is adaptive spiking

**else if** *V*_g2_ > *V*_g2,symbol_^+^(*V*_g1_), **then**

Mode is adaptive spiking

**else**

Mode is fast-spiking

**else**

Simulate neuron dynamics, extract ISI

*ε* = 0.05	// Threshold for adaptation

**if** (ISI[-1] – ISI[0]) / ISI[0] > *ε*, **then**

Mode is adaptive spiking	// ISI increased significantly

**else**

Mode is fast-spiking

**Supporting Note 3: Area overhead, scalability and power consumption of the neuron**

To facilitate the evaluation of the area overhead and energy consumption, we constructed the reconfigurable neuron in SPICE using the GlobalFoundries 180 nm open-source process^[5]^. Table S1 lists a set of circuit parameters with which the neuron can function correctly. This set of parameters were also used to investigate the effect of hardware nonidealities on neuron behaviors, which is detailed in Supporting Notes 4 and 5.

Based on the reported area overhead for various standard cells in the process node library, especially the area overhead of inverters of different drive strengths, we estimated and extrapolated that the area needed to fit *M*_1_, *M*_2_, *M*_3_ and *M*_4_ is approximately 40.51 μm^2^. The area needed to fit *R*_1_ assuming constant cell height is approximately 17.56 μm^2^. The combined area of *C*_1_ and *C*_2_ is 15500 μm^2^. The VO_2_ device does not introduce additional area overhead if fabricated at the back-end-of-line (BEOL). Hence, the total area of the neuron circuit is approximately 15558 μm^2^, which is dominated by the area of the capacitors. Nevertheless, given the dynamical equations governing the control module (Equation 10 in Supporting Note 1), further area optimizations can be made by scaling down *C*_1_ and *C*_2_ while simultaneously scaling down the current through *M*_1_, *M*_2_ and *M*_3_ by the same factor to ensure an invariant evolution of *V*_ctrl1_ and *V*_ctrl2_. For instance, if *C*_1_ and *C*_2_ is reduced by 10 times, the channel lengths of *M*_1_, *M*_2_ and *M*_3_ need to be increased by 10 times. In this case, the area needed to fit *M*_1_, *M*_2_, *M*_3_ and *M*_4_ is approximately 273 μm^2^, while the combined area of *C*_1_ and *C*_2_ is 1550 μm^2^. Hence, the total area of the neuron circuit is greatly reduced to 1823 μm^2^.

The scalability of the neuron towards newer process nodes depends on the VO_2_ memristor. In particular, circuits implemented using newer process nodes operate at lower supply voltages, which in turn requires reducing *V*_th,VO2_ to ensure that the neuron can reach its firing threshold. This can be achieved by reducing the gap between the two electrodes^[6]^ and enhancing heat confinement via thermal engineering to facilitate the insulator-metal transition of VO_2_^[7]^. Moreover, as aforementioned, scaling down *C*_1_ and *C*_2_ requires simultaneously reducing the drive strength of the transistors. Besides increasing the length of the transistors, the drive strength can also be reduced by introducing a load resistor or a diode-connected transistor between the source terminal of *M*_1_ and *V*_dd_. Although this strategy introduces an additional device, it obviates the need to design transistors with extremely large channel lengths, which could be further beneficial in reducing area overhead and increasing scalability

The power consumption of the neuron depends on the biasing of the inputs and the operating regime. We obtained representative energy per spike (*E*_sp_) and average power of the integrator (*P*_avg_) figures for each regime through simulations and listed them in Table S2. In our simulations, we used a current input as the main input stimulus of the neuron, hence the energy consumption of the integration module is *E*_int_ = ∫ *I*_in_×*V*_mem_ d*t*. The energy consumption of the control module is *E*_ctrl_ = ∫ *I*_supply_×*V*_dd_ d*t*, where *I*_supply_ is the instantaneous current provided by the power source and the energy figure takes into account the initial charging of *C*_1_ and *C*_2_ to *V*_dd_ before *I*_in_ is fed into the neuron. *E*_sp_ is therefore the sum of *E*_int_ and *E*_ctrl_, divided by the total number of spikes (*N*). The energy consumption was integrated up to the firing of the sole spike in the single-spiking regime and up to the firing of the last spike in the phasic bursting regime. In the adaptive spiking and fast-spiking regimes, the energy consumption was integrated up to the firing of the final spike in a 1 ms duration. As can be seen, *E*_ctrl_ is negligible compared to *E*_int_. In terms of the energy per spike, the adaptive spiking regime has the largest figure, while the other three modes have much lower figures. This is due to the significantly lower spiking frequency during adaptation. *P*_avg_ is *E*_int_ divided by the integration time.

Note that the circuit is unoptimized for low energy or power in its current configuration. Firstly, the capacitance of the connecting wires and the source measurement unit make up the parasitic *C*_3_ in our experimental setup, which can be as large as 1 nF. On the other hand, the intrinsic capacitance of VO_2_ memristors ranges from pF to sub-100 fF depending on the operating frequency^[8]^. Integrating the VO_2_ memristor at the BEOL directly atop the rest of the circuit and driving the neuron using sources such as a current mirror with much lower output capacitance can drastically reduce the parasitic *C*_3_. Assuming a conservative 100× reduction in *C*_3_, the temporal dynamics of the integration module becomes 100× faster, resulting in a 100× lower *E*_int_ in the single-spiking, phasic bursting and the fast-spiking regimes. By simultaneously scaling the capacitors and transistors in the control module such that the slower adaptive dynamics also speeds up by the same amount, the *E*_int_ in the adaptive spiking regime will also reduce by 100×. Moreover, optimizations in the geometry and fabrication of the VO_2_ memristor to lower *V*_th,VO2_ will further reduce *P*_avg_ and *E*_int_ simultaneously. The possibility to then use more advanced process nodes with a lower *V*_dd_ will also reduce *E*_ctrl_.

For the sake of completeness, we also investigated the static power consumption of the neuron. In this case, we assumed no input to the integrator such that the static power arises solely from the control module in idle. We simulated the control module to obtain the quiescent current through the transistors for power calculations. Besides, we set both *V*_g1_ and *V*_g2_ to either *V*_dd_ or 0 V to find the static power consumption under two extremes. In our hardware demonstration, we used the off-the-shelf transistors, which have large off-state leakage currents. When *V*_g1_ and *V*_g2_ are both biased at a *V*_dd_ of 10 V, the quiescent current is about 10 pA, and the static power consumption is 100 pW. When *V*_g1_ and *V*_g2_ are both biased at 0 V, the quiescent current is about 250 nA, and the static power consumption is 2.5 μW. If the neuron is designed using the GlobalFoundries 180 nm process node, the off-state leakage currents are much lower. When *V*_g1_ and *V*_g2_ are both biased at a *V*_dd_ of 6 V, the quiescent current is about 0.01 fA, and the static power consumption is 0.06 fW. When *V*_g1_ and *V*_g2_ are both biased at 0 V, the quiescent current is about 16.4 pA, and the static power consumption is 98.4 pW.

**Supporting Note 4: Effect of *V*_th,VO2_ variations on regime boundaries**

This section analyzes how shifts in *V*_th,VO2_ shifts the four boundaries between operating regimes (black solid lines in the phase diagram in Figure 2h).

Boundaries (i)-(ii): Determining how these boundaries shift requires analyzing how *T*_ini_ and *T*_sat_ varies with *V*_th,VO2_. *T*_ini_ increases when *V*_th,VO2_ is larger because the rising time and falling time of *V*_mem_ within one spiking period increases (Equations 6-7 in the main text). On the other hand, *T*_sat_ also increases when *V*_th,VO2_ is larger. Firstly, the spike amplitude across *R*_1_ increases, resulting in a larger *I*_ds,_*_M_*_3_. Secondly, *M*_3_ closes for a longer duration since the time it takes for *V*_mem_ to fall from *V*_th,VO2_ to *V*_th,_*_M_*_3_ increases. The combined effect is therefore a larger Δ*V*_sp_ and *T*_sat_ (first equality of Equations 1-2 in the main text). Note that *T*_sat_ is linearly proportional to Δ*V*_sp_ and also *I*_ds,_*_M_*_3_, which in turn is affected by *V*_th,VO2_ quadratically assuming saturation, but *T*_ini_ is affected by *V*_th,VO2_ logarithmically. Therefore, *T*_sat_ increases faster than *T*_ini_. To maintain *T*_sat_ = *T*_ini_, |*I*_ds,_*_M_*_1,sat_| and |*I*_ds,_*_M_*_2,sat_| need to increase, which means that the boundaries shift toward lower *V*_g1_ and *V*_g2_. We performed additional simulations to verify that the circuit does indeed transition from fast-spiking to adaptation at lower *V*_g1_ (Figure S3) and *V*_g2_ (Figure S4) values when *V*_th,VO2_ increases.

Boundary (iii): Equation 3 in the main text does not involve *V*_th,VO2_. Therefore, variations in *V*_th,VO2_ do not shift this boundary .

Boundary (iv): Δ*V*_sp_ and *V*_ctrl2,th_ both decrease when *V*_th,VO2_ is smaller. Based on Equation 4 in the main text, *V*_g2_ increases. Therefore, this boundary shifts towards larger *V*_g2_. On the contrary, this boundary shifts towards smaller *V*_g2_ when *V*_th,VO2_ is larger. However, this shift tends to be small as *C*_1_ is usually set to be much smaller than *C*_2_, and their ratio scales down the effect of variations in *V*_th,VO2_ significantly.

**Supporting Note 5: Effect of hardware nonidealities on spike coding**

Various factors such as *V*_g1,2_ noise, *V*_dd_ noise, CMOS process variations, VO_2_ memristor variations, short-channel effects and parasitic effects affect the accuracy and stability of the voltage evolution of *C*_1_ and *C*_2_. Such deviations are reflected in the output spike trains as deviations in the spike train parameters from the ideal values, ultimately affecting the encoding results.

For *V*_g1,2_ noise, *V*_dd_ noise, CMOS process variations and VO_2_ memristor variations, we performed Monte Carlo analyses based on SPICE simulation to investigate how they affect the spike train parameters. For each set of circuit parameter and level of noise or variations, we performed *n* = 10 independent simulation runs, extracted the spike train parameters and evaluated the coefficient of variation (*C*_v_) of each parameter across the runs. We simulated only the adaptive spiking and phasic bursting regimes as *f*_sp_ and *t*_sp_ essentially measure the same quantity as *T*_ini_ and *T*_burst_, and thus variations in *T*_ini_ and *T*_burst_ can reflect variations in *f*_sp_ and *t*_sp_ . The extraction of *T*_in_, *T*_burst_, *T*_sat_ and *N*_sp_ from the simulated spiking waveforms were relatively straightforward. As for the extraction of *N*_o_, we counted the number of spikes up to the point when the increase in the ISI exceeds 10% of (*T*_sat_ - *T*_ini_). For short-channel effects and parasitic effects, we provide quantitative analyses based on estimations instead.

The variations in adaptive spiking parameters in response to *V*_g1_ noise are summarized in Figure S12a-c. Since *T*_ini_ is independent of *V*_g1_, *V*_g1_ noise does not lead to *T*_ini_ variations. *N*_o_ and *T*_sat_ are dependent on *V*_g1_ (Equations 1-2 and 11 in the main text), and thus *V*_g1_ noise causes *N*_o_ and *T*_sat_ variations. *T*_sat_ becomes increasingly sensitive to *V*_g1_ at larger *V*_g1_ values (Figure 3h), which explains the increasing *C*_v,_*_T_*_sat_ with *V*_g1_. However, *N*_o_ is only weakly dependent on *V*_g1_, and thus *C*_v,_*_N_*_o_ is not significantly dependent on *V*_g1_. On the other hand, the variations in adaptive spiking parameters and phasic bursting parameters in response to *V*_g2_ noise are summarized in Figure S12d-f and g-h, respectively. Firstly, *N*_o_ and *N*_sp_ encodes *V*_g2_, therefore *V*_g2_ noise translates into *N*_o_ and *N*_sp_ variations. Specifically, mean *N*_o_ and *N*_sp_ reduce with increasing *V*_g2_, resulting in rapidly increasing *C*_v,_*_N_*_o_ and *C*_v,_*_N_*_sp_. Secondly, *C*_v,_*_T_*_ini_ and *C*_v,_*_T_*_burst_ increase only at large *V*_g2_. This is because at small *V*_g2_, *N*_o_ and *N*_sp_ is large and the initial spiking period depends only on the integrator module, whereas at large enough *V*_g2_, the onset of adaptation and the final elongated spiking period towards the end of the burst happens almost immediately after the first spike, causing *T*_ini_ and *T*_burst_ to become dependent on *V*_g2_. Lastly, since *V*_g2_ also modulates *T*_sat_ at large enough values (Figure 3h), *C*_v,_*_T_*_sat_ also increases at large *V*_g2_, albeit maintaining at a level lower than *C*_v,_*_T_*_ini_ under *V*_g2_ noise or *C*_v,_*_T_*_sat_ under *V*_g1_ noise.

The variations in spike train parameters in response to *V*_dd_ noise are summarized in Figure S13. When varying *V*_g1_ in adaptive spiking, *T*_ini_ exhibits no significant variations. *N*_o_ also exhibits no significant variations at larger *V*_g1_. The increase in *C*_v,_*_N_*_o_ towards smaller *V*_g1_ can be attributed to the decreased difference between *T*_sat_ and *T*_ini_, which causes the extraction of *N*_o_ by the 10% threshold method to become increasingly sensitive to variations in ISI. *C*_v,_*_T_*_sat_ increases with *V*_g1_, which, as aforementioned, is due to *T*_sat_ being increasingly sensitive to |*V*_gs,_*_M_*_1_| at larger *V*_g1_ values. When varying *V*_g2_ in adaptive spiking, *N*_o_ exhibits no significant variations, since unlike *V*_g1_, *V*_g2_ cannot lower *T*_sat_. *C*_v,_*_T_*_ini_ is insignificant at lower *V*_g2_ and increases at larger *V*_g2_ for the same reason given in the discussion on the effect of *V*_g1,2_ noise. *C*_v,_*_T_*_sat_ does not depend on *V*_g2_ significantly as the variations in |*V*_gs,_*_M_*_1_| cannot be isolated and is the dominant cause of variations in *T*_sat_. The analyses for *C*_v,_*_N_*_sp_ and *C*_v,_*_T_*_burst_ in phasic bursting when *V*_g2_ is varied are the same as that for *C*_v,_*_N_*_o_ and *C*_v,_*_T_*_ini_.

The variations in spike train parameters in response to VO_2_ memristor variations are summarized in Figure S14. In particular, we considered variations in *V*_th,VO2_ as it affects more parts of the neuron circuit and to a larger extent than variations in other VO_2_ parameters. For instance, an increase in *V*_th,VO2_ increases the intrinsic spiking period of the integrator module, increases Δ*V*_sp_ due to a larger *I*_ds,_*_M_*_4_ caused by a larger spike voltage and increases *V*_ctrl2,th_. Thus, *T*_ini_, *T*_burst_ and *T*_sat_ increase, while *N*_o_ and *N*_sp_ decrease. Several observations can be made from Figure S14. Firstly, the level of variations in spike train parameters do not significantly change when *V*_g1_ is varied. Secondly, *C*_v,_*_N_*_o_ and *C*_v,_*_T_*_ini_ generally increases with *V*_g2_ while *C*_v,_*_T_*_sat_ does not vary significantly with *V*_g2_. Besides, at similar levels of *C*_v,_*_V_*_th,VO2_, *C*_v,_*_N_*_o_ is generally larger than *C*_v,_*_T_*_sat_ and *C*_v,_*_T_*_ini_, indicating that *N*_o_ is the most sensitive to variations in *V*_th,VO2_. In phasic bursting, *C*_v,_*_N_*_sp_ does not seem to be significantly dependent on *V*_g2_ while *C*_v,_*_T_*_ini_increases with *V*_g2_.

The variations in spike train parameters in response to CMOS process variations are summarized in Figure S15. Here, we utilized the process variation parameters and switches provided in the GlobalFoundries 180 nm SPICE model^[5]^ to perform simulations with nonideal process parameters. Several observations can be noted. Firstly, *C*_v,_*_N_*_o_ and *C*_v,_*_T_*_ini_ when *V*_g1_ is varied in adaptive spiking, *C*_v,_*_T_*_sat_ when *V*_g1_ or *V*_g2_ is varied, and *C*_v,_*_T_*_burst_ when *V*_g2_ is varied in phasic bursting do not change significantly. *C*_v,_*_N_*_o_ , *C*_v,_*_T_*_ini_ and *C*_v,_*_T_*_ini_ generally increase with *V*_g2_. These observations are similar to that under variations in *V*_th,VO2_. Nevertheless, variations in *T*_ini_ at any *V*_g1_ and at low enough *V*_g2_ as well as variations in *T*_burst_ at low enough *V*_g2_ are marginal. Such increase in the level of variations at larger *V*_g2_ is again due to the onset of adaptation and the elongation of the spiking period towards the end of the burst happening almost immediately after the first spike.

Next, we analyze how short-channel effects, such as channel length modulation and leakage might affect spike encoding. With channel length modulation, *I*_ds_ no longer saturates at large *V*_ds_, but increases with it. This shortens the charging time of *C*_1_ and *C*_2_, therefore decreasing *T*_sat_ for any given combination of *V*_g1_ and *V*_g2_. Besides, the regime boundaries between fast-spiking and adaptive spiking also shift towards larger *V*_g1_ and *V*_g2_. As for the increased off-state leakage current due to short channel, firstly, the leaky *M*_3_ slows down the charging of *C*_2_ during adaptation, therefore increasing *T*_sat_. However, since the off-state leakage current at near-zero *V*_gs_ is still orders of magnitude smaller than *I*_ds_ near the threshold^[9]^, the effect of off-state leakage current on *T*_sat_ is negligible. Secondly, when the neuron in the single-spiking mode or the bursting mode enters the resting state, the leakage current in *M*_1_, *M*_2_ and *M*_3_ might together alter the equilibrium voltage level of *C*_1_ and *C*_2_, which might subsequently lead to persistent firing if the net current is such that it charges the capacitors and raises *V*_ctrl2_. The leakage current in *M*_4_ might also raise *V*_mem_ slowly even when the neuron is in the resting state. However, if given an *I*_off_ of ~1 fA (assuming *V*_th_ ≈ 300 mV at *I*_off_ = 100 pA/μm, a subthreshold slope of 70 mV/decade, and a width of 0.1 μm)^[10]^, the Δ*V*/Δ*t* of an aggressively scaled down 10 fF capacitor is only as high as 0.1 V/s, which is on a much larger timescale compared to the dynamics of the neuron. Therefore, we can conclude that the effects of transistor off-state leakage current on the spiking behavior of the neuron and, subsequently, the network performance are negligible. We would like to stress that one possible strategy to optimize scalability is by reducing the capacitor values while increasing the length of the transistors (see Supporting Note 3). An additional benefit of this is the suppression of short channel effects, which not only further eensures proper neuron operation, but also reduces the complexity of the mathematical model used for encoding

Finally, we analyze how parasitic capacitances and parasitic source/drain resistances might affect spike encoding and network performance. The significance of these parasitic elements depends on the sizes of the capacitors in the control module. The increase in *C*_2_ due to parasitic capacitances will slow down its charging during adaptation, thus increasing *T*_sat_. Besides, *N*_o_ and *N*_sp_ are also affected in such a way that if the ratio *C*_1_/*C*_2_ decreases due to parasitics, *N*_o_ and *N*_sp_ decreases, and vice versa. However, the changes in *T*_sat_, *N*_o_ and *N*_sp_ can be marginal. Taking FinFETs or lateral nanowire transistors as examples, the total parasitic capacitance per fin is less than 100 aF^[11]^. Since transistors with low drive strength is needed if *C*_1_ and *C*_2_ are scaled down, fin depopulation can be assumed. In this case, even an aggressively scaled *C*_2_ of 10 fF is still two orders of magnitude larger than the parasitic capacitance. On the other hand, parasitic source/drain resistances reduce the drive strength of the transistors, which increases *T*_sat_ and decreases *N*_o_. Nevertheless, the reduction in drive strength is exactly what is needed in the control module to accompany the scaling down of *C*_1_ and *C*_2_. This then relaxes the need to substantially increase the channel length of the transistors, which is further beneficial for scalability. The caveat is that the exact mathematical model of the encoding strategies will become more complex, and empirical models might then be useful instead.

**Supporting Note 6: Effect of hardware nonidealities on network performance**

We performed inference runs while introducing varying levels of different nonidealities into the trained networks presented in the main text. For each type of nonideality, the relevant circuit parameters were randomly sampled from Gaussian distributions in which the ideal parameter value and the spread of the nonideal parameter are the mean and standard deviation, respectively, of the corresponding distribution. After random sampling, the circuit parameters remained constant within one inference run. This effectively models device-to-device variations in addition to drifts in device parameters, power supply and biasing voltages over a period of time longer than the time it takes for one inference.

In the hazard avoidance task, we introduced hardware nonidealities only in the output neuron. We performed inference on the test dataset for *n* = 10 independent times for each level of variations or noise We compared the spike train parameters of the nonideal output neuron with that of the target output obtained with an ideal neuron. The mean test accuracies of driving actions and the mean squared relative errors (MSRE) of the spike train parameters are summarized in Figure S16. For *C*_v,_*_V_*_th,VO2_ ≤ 5%, *σ_V_*_th,MOS_ ≤ 60 mV, *σ_V_*_g1,2_ ≤ 100 mV, and *σ_V_*_dd_ ≤ 200 mV, the nonideal output neuron can successfully spike in the correct mode every time, maintaining a 100% accuracy in the generated driving actions. Even for levels of variations larger than those typically encountered in the real world, the degradation in accuracy is small. Besides, the MSREs of the spike train parameters increase when the level of each type of nonideality increases. Similarly, for levels of variations typically encountered in the real world, low errors can still be achieved. For VO_2_ memristors in particular, *C*_v,_*_V_*_th,VO2_ values as low as ~4% have been reported, which can ensure low coding errors^[8]^.

For the heterogeneous LSNN and the dynamic LSNN, we investigated the effect of nonideal hidden layer neurons on the inference process in the EPHNOGRAM task. We introduced varying degrees of hardware nonidealities during inference and performed classification on the test dataset for *n* = 10 independent times for each level of variations or noise. The mean test accuracies of the heterogeneous LSNN and the dynamic LSNN are summarized in Figures S20 and S21, respectively. While the mean test accuracies degrade with increasing levels of each type of nonideality, both types of LSNN exhibit different sensitivity to different nonidealities. The mean accuracy achieved by the heterogeneous LSNN degrades more under *V*_g1,2_ noise and *V*_dd_ noise, while the mean accuracy achieved by the dynamic LSNN degrades more under *V*_g1,2_ noise and VO_2_ variations. Overall, the dynamic LSNN is more robust than the heterogeneous LSNN, especially under CMOS process variations, *V*_g1,2_ noise and *V*_dd_ noise. A high accuracy can still be maintained under levels of variations typically encountered in the real world.

**References**

[1]	Cell Features :: Allen Brain Atlas: Cell Types, https://celltypes.brain-map.org/data.

[2]	Y. Yang, F. Zhu, X. Zhang, et al., *Nature Communications* **2024**, *15*, 4318.

[3]	Y. Xiao, Y. Liu, B. Zhang, et al., *Science Advances* **2025**, *11*, eadr6733.

[4]	F. Li, D. Li, C. Wang, et al., *Nature Communications* **2024**, *15*, 3689.

[5]	GlobalFoundries 0.18UM 3.3V/(5V)6V MCU PDK documentation, https://gf180mcu-pdk.readthedocs.io/.

[6]	R. Yuan, Q. Duan, P. J. Tiw, et al., *Nature Communications* **2022**, *13*, 3973.

[7]	P. Chen, X. Zhang, J. Qiu, et al., *Advanced Functional Materials* **2025**, 2423800.

[8]	P. J. Tiw, R. Yuan, T. Zhang, L. Yu, Y. Yang, *Nature Electronics* **2026**, *9*, 225.

[9]	R. Xie, P. Montanini, K. Akarvardar, et al., in *2016 IEEE International Electron Devices Meeting (IEDM)*, **2016**, p. 2.7.1-2.7.4.

[10]	IEEE International Roadmap for Devices and Systems, *More Moore*, Institute of Electrical and Electronics Engineers **2017**.

[11]	M. G. Bardon, Y. Sherazi, P. Schuddinck, et al., in *2016 IEEE International Electron Devices Meeting (IEDM)*, **2016**, p. 28.2.1-28.2.4.
